# Whole‐exome sequencing for variant discovery in blepharospasm

**DOI:** 10.1002/mgg3.411

**Published:** 2018-05-16

**Authors:** Jun Tian, Satya R. Vemula, Jianfeng Xiao, Enza Maria Valente, Giovanni Defazio, Simona Petrucci, Angelo Fabio Gigante, Monika Rudzińska‐Bar, Zbigniew K. Wszolek, Kathleen D. Kennelly, Ryan J. Uitti, Jay A. van Gerpen, Peter Hedera, Elizabeth J. Trimble, Mark S. LeDoux

**Affiliations:** ^1^ Departments of Neurology and Anatomy and Neurobiology University of Tennessee Health Science Center Memphis Tennessee; ^2^ Department of Neurology Second Affiliated Hospital School of Medicine Zhejiang University Hangzhou Zhejiang China; ^3^ Department of Molecular Medicine University of Pavia Pavia Italy; ^4^ Neurogenetics Unit IRCCS Santa Lucia Foundation Rome Italy; ^5^ Department of Basic Clinical Sciences, Neuroscience and Sense Organs Aldo Moro University of Bari Bari Italy; ^6^ Department of Medical Sciences and Public Health University of Cagliari Cagliari Italy; ^7^ Department of Neurology and Psychiatry Sapienza University of Rome Rome Italy; ^8^ Department of Neurology Faculty of Medicine Medical University of Silesia Katowice Poland; ^9^ Department of Neurology Mayo Clinic Florida Jacksonville Florida; ^10^ Department of Neurology Vanderbilt University Nashville Tennessee

**Keywords:** blepharospasm, cerebellum, dystonia, Purkinje cell, whole‐exome sequencing

## Abstract

**Background:**

Blepharospasm (BSP) is a type of focal dystonia characterized by involuntary orbicularis oculi spasms that are usually bilateral, synchronous, and symmetrical. Despite strong evidence for genetic contributions to BSP, progress in the field has been constrained by small cohorts, incomplete penetrance, and late age of onset. Although several genetic etiologies for dystonia have been identified through whole‐exome sequencing (WES), none of these are characteristically associated with BSP as a singular or predominant manifestation.

**Methods:**

We performed WES on 31 subjects from 21 independent pedigrees with BSP. The strongest candidate sequence variants derived from *in silico* analyses were confirmed with bidirectional Sanger sequencing and subjected to cosegregation analysis.

**Results:**

Cosegregating deleterious variants (GRCH37/hg19) in *CACNA1A* (NM_001127222.1: c.7261_7262delinsGT, p.Pro2421Val)*, REEP4* (NM_025232.3: c.109C>T, p.Arg37Trp), *TOR2A* (NM_130459.3: c.568C>T, p.Arg190Cys)*,* and *ATP2A3* (NM_005173.3: c.1966C>T, p.Arg656Cys) were identified in four independent multigenerational pedigrees. Deleterious variants in *HS1BP3* (NM_022460.3: c.94C>A, p.Gly32Cys) and *GNA14* (NM_004297.3: c.989_990del, p.Thr330ArgfsTer67) were identified in a father and son with segmental cranio‐cervical dystonia first manifest as BSP. Deleterious variants in *DNAH17*,*TRPV4*,*CAPN11*,*VPS13C*,*UNC13B*,*SPTBN4*,*MYOD1*, and *MRPL15* were found in two or more independent pedigrees. To our knowledge, none of these genes have previously been associated with isolated BSP, although other *CACNA1A* mutations have been associated with both positive and negative motor disorders including ataxia, episodic ataxia, hemiplegic migraine, and dystonia.

**Conclusions:**

Our WES datasets provide a platform for future studies of BSP genetics which will demand careful consideration of incomplete penetrance, pleiotropy, population stratification, and oligogenic inheritance patterns.

## 1 INTRODUCTION

Dystonia is defined as a movement disorder characterized by sustained or intermittent muscle contractions causing abnormal, often repetitive, movements, postures, or both (Albanese et al., 2013). In general, adult‐ or late‐onset dystonia without evidence of overt degeneration or structural lesions of the nervous system is referred to as isolated dystonia and can be inherited in an autosomal‐dominant fashion with reduced penetrance. The most common forms of focal dystonia are cervical dystonia and blepharospasm (BSP). Blepharospasm (BSP) (OMIM: 606798) is characterized by involuntary orbicularis oculi spasms that are usually bilateral, synchronous, and symmetrical (Defazio et al., 2015). Review of BSP epidemiological data provides prevalence estimates ranging from 16 to 133 per million (Defazio, Abbruzzese, Livrea, & Berardelli, 2004). BSP is significantly more common in females (>2F:1M) with a mean age of onset at approximately 55 years (O'Riordan et al., 2004). In comparison to cervical and laryngeal dystonia, BSP is more likely to spread to other body parts (Weiss et al., 2006). Most commonly, BSP spreads to contiguous craniocervical segments (lower face, masticatory muscles, and neck). The term segmental craniocervical dystonia is applied to the combination of BSP and dystonia of other head and neck muscles (LeDoux, 2009). Herein, BSP‐plus (BSP+) will be used to denote subjects with BSP who exhibit subsequent spread to other anatomical segments (LeDoux, 2009; Waln & LeDoux, 2011). Sensory tricks or geste antagonistes are highly specific to dystonia, reported in a high percentage of patients with BSP, and can facilitate the diagnosis of BSP (Defazio, Hallett, Jinnah, & Berardelli, 2013). However, without valid genetic biomarkers, the diagnosis of BSP can be difficult, even for experienced clinicians (Defazio et al., 2013).

Although rare cases of isolated BSP have been linked to *THAP1* (OMIM 609520) mutations (LeDoux et al., 2012; Vemula et al., 2014), the genetic underpinnings of this focal dystonia remain largely unknown. In one study, 233 relatives of 56 probands with primary BSP were examined and 27% had a first‐degree relative affected by BSP or other dystonia (Defazio, Martino, Aniello, Masi, Abbruzzese, et al., 2006). Using an autosomal dominant model, penetrance was approximately 20% in pedigrees with BSP (Defazio, Martino, Aniello, Masi, Abbruzzese, et al., 2006; Defazio, Martino, Aniello, Masi, Gigante, et al., 2006). For comparison, penetrance of the classic ΔGAG mutation in *TOR1A* (OMIM 605204, DYT1) is 30%–40% (Bressman et al., 2000). Approximately 10% of subjects in large biorepositories of isolated dystonia have a first‐ or second‐degree relative with dystonia (LeDoux et al., 2016; Vemula et al., 2013, 2014; Xiao et al., 2010, 2011, 2012). Even though late‐onset isolated dystonia has a considerable “heritable” component, large pedigrees adequately powered for linkage analysis are rare. Conversely, small multiplex pedigrees with 2 or 3 affected individuals are not uncommon.

In six published clinical series, 1st‐degree relatives of probands with isolated dystonia were subjected to examination (Defazio, Livrea, Guanti, Lepore, & Ferrari, 1993; Defazio, Martino, Aniello, Masi, Abbruzzese, et al., 2006; Leube, Kessler, Goecke, Auburger, & Benecke, 1997; Stojanovic, Cvetkovic, & Kostic, 1995; Waddy, Fletcher, Harding, & Marsden, 1991). Within these reported families, overall phenotypic concordance‐discordance was approximately 50%–50%. However, discordant pedigrees are relatively more common in probands with BSP than cervical dystonia (Defazio, Berardelli, & Hallett, 2007). An example of phenotypic discordance would be the presence of BSP in a proband and cervical dystonia in one of the proband's siblings. Phenotype concordance is the presence of a single anatomical distribution of dystonia (e.g., BSP) in all affected family members.

Herein, we report the results of whole‐exome sequencing (WES) of 31 subjects from 21 independent pedigrees with BSP and/or BSP+, the largest collection of BSP pedigrees examined to date. Our series includes both concordant and discordant pedigrees. Our results will facilitate a better understanding of the genetic underpinnings of isolated BSP and other, mainly adult‐onset, dystonias. A collection of *in silico* tools, including dbNSFP (Dong et al., 2015; Liu, Jian, & Boerwinkle, 2011; Liu, Wu, Li, & Boerwinkle, 2016), dbscSNV (Jian, Boerwinkle, & Liu, 2014), Combined Annotation‐Dependent Depletion (CADD; Kircher et al., 2014), REVEL (Ioannidis et al., 2016), and MutationTaster (Schwarz, Cooper, Schuelke, & Seelow, 2014) were used to identify and prioritize candidate sequence variants. Putative disease‐associated variants were confirmed with bidirectional Sanger sequencing, followed by cosegregation analysis. Cosegregating deleterious variants in *CACNA1A* (OMIM 601011), *REEP4* (OMIM 609349)*, TOR2A* (OMIM 608052), *ATP2A3* (OMIM 601929), *HS1BP3* (OMIM 609359), *GNA14* (OMIM 604397) and *DNAH17* (OMIM 610063) were identified in single pedigrees.

## 2 MATERIALS AND METHODS

### 2.1 Ethical compliance

All human studies were conducted in accordance with the Declaration of Helsinki with formal approval from the University of Tennessee Health Science Center Institutional Review Board (IRB; 01‐07346‐FB, 05‐08331‐XP, and 14‐03320‐XP) and ethics committees of all participating centers. All subjects gave written informed consent for genetic analyses and disclosure of medical information.

### 2.2 Subjects

Subjects in this study were examined by at least one neurologist with subspecialty expertise in movement disorders. Subjects were asked to perform specific tasks, including holding their eyes open, opening and closing their eyes gently, opening and closing their eyes forcefully, along with additional verbal and postural maneuvers designed to capture masticatory, laryngeal or cervical involvement. A clinical diagnosis of definite BSP was given to subjects that exhibited increased blinking and stereotyped, bilateral and synchronous orbicularis oculi spasms inducing narrowing/closure of the eyelids (Defazio et al., 2013). Subjects with isolated episodes of increased eyelid blinking were given a diagnosis of possible BSP. Each affected or possibly affected family member was queried for the presence of sensory tricks. WES was completed on a total of 31 subjects from 21 pedigrees from the United States, Poland, and Italy (Table 1). Prior to WES, pathogenic variants in *THAP1*,* GNAL* (OMIM 139312) and Exon 5 of *TOR1A* were excluded as previously described (LeDoux et al., 2012; Vemula et al., 2013; Xiao et al., 2009, 2010). Two pedigrees were African–American and 19 pedigrees were Caucasian of European descent. The results of WES on the proband of African–American pedigree 10908 were previously reported (Xiao, Thompson, Vemula, & LeDoux, 2016) and deposited in Sequence Read Archive (SRX1790848).

**Table 1:** BSP and BSP+ subjects examined with whole‐exome sequencing

Subject	Age	Age of onset	Sex	Ethnicity	BSP family history	Anatomical distribution	Select candidate genes
10012	77	60	F	Caucasian	No	Segmental dystonia (BSP, oromandibular, lower face, cervical)	*KCNH4, CHRNA7, SPTBN4, ATP13A2*
10014	70	47	F	Caucasian	No	Segmental dystonia (BSP, oromandibular, lower face)	*KCNG4, PLP1, KCNS1, ACLY, VPS13C*
10035	67	55	F	Caucasian	No	Segmental dystonia (BSP, oromandibular, lower face, cervical)	*TRPV4, TBP, IMP4, UBXN4*
10036	69	66	F	Caucasian	No	Segmental dystonia (BSP, cervical)	*HK1, PRUNE2, NUMBL, MRPL15*
10043‐I‐1	83	57	M	Caucasian	Yes	Segmental dystonia (BSP, oromandibular, lower face, cervical)	*GNA14, HS1BP3, NEFH, RWDD2A*
10043‐II‐2	51	45	M	Caucasian	Yes	Segmental dystonia (BSP, pharyngeal, laryngeal, cervical), Parkinsonism	*GNA14. HS1BP3, NEFH, RWDD2A*
10064	60	47	M	Caucasian	Yes	Segmental dystonia (BSP, oromandibular, lower face, cervical)	*HECW2, CDH4, RABL2B, AP4B1, SCN3A*
10076	62	61	F	Caucasian	No	Segmental dystonia (BSP, cervical)	*CAPN11, REEP2, MYO1B, DNAH17, ATP13A2*
10178	59	20	M	Caucasian	Yes	BSP	*ZZEF1, KCNA5, MUYOD1, MRPL15*
10193	77	69	F	Caucasian	Yes	BSP	*IGSF21, MYOD1*
10274‐II‐3	56	45	M	AA	Yes	Segmental dystonia (BSP, cervical)	*TRPV4, WDFY3, ZFYVE9*
10274‐II‐6	50	50	F	AA	Yes	BSP	*TRPV4, WDFY3, ZFYVE9*
10455	58	48	F	Caucasian	Yes	Segmental dystonia (BSP, oromandibular, lower face, cervical)	*CADPS, SNPH, ATP2B1, SLC12A2, CAPN11, VSP13DC, SPTBN4, BTNL3*
10908‐II‐3	66	48	M	AA	Yes	Segmental dystonia (BSP, oromandibular, lower face, cervical)	*REEP4*
10908‐III‐9	33	30	M	AA	Yes	BSP	*REEP4*
25056	70	59	F	Caucasian	Yes	Segmental dystonia (BSP, oromandibular, lower face, arm tremor)	*ABCA2, MYT1L*
25069	61	56	M	Caucasian	Yes	BSP (with arm tremor)	*LRP1B, PCDHGA3, LAMA1, UNC13B, ATP13A2*
25215	57	54	F	Caucasian	Yes	BSP (with arm tremor)	*AGAP1, EPS15L1, SCN1A, UNC13B, TOP3B*
45263	78	77	M	Caucasian	Yes	BSP	*INO80, DNAH17*
85020	66	50	F	Caucasian	Yes	BSP	*LRP1, GCH1, DDHD2, UNK*
NG0362‐II‐2	57	39	M	Caucasian	Yes	BSP	*CACNA1A*
NG0362‐I‐1	76	67	M	Caucasian	Yes	BSP	*CACNA1A*
NG0362‐III‐1	35	NA	M	Caucasian	Yes	BSP	*CACNA1A*
NG0369‐II‐2	80	58	F	Caucasian	Yes	BSP	*TOR2A, PCDH15, GTDC1*
NG0369‐III‐2	52	NA	F	Caucasian	Yes	BSP	*TOR2A, PCDH15, GTDC1*
NG0369‐III‐6	46	NA	F	Caucasian	Yes	BSP	*TOR2A, PCDH15, GTDC1*
NG0450‐IV‐3	80	53	F	Caucasian	Yes	BSP	*TRPV4, SERPINB9, CNTNAP2*
NG0450‐V‐4	64	40	F	Caucasian	Yes	BSP	*TRPV4, SERPINB9, CNTNAP2*
NG0450‐V‐6	51	38	M	Caucasian	Yes	Writer's cramp	*TRPV4, SERPINB9, CNTNAP2*
NG1072‐II‐5	72	NA	M	Caucasian	Yes	BSP	*ATP2A3*
NG1072‐IV‐2	24	21	F	Caucasian	Yes	Cervical dystonia	*ATP2A3*

AA, African–American; NA, not available.

### 2.3 Whole‐exome sequencing

The concentration and quality of genomic DNA (gDNA) extracted from peripheral blood were examined with a NanoDrop^®^ ND‐1000 (Thermo Scientific), the Qubit^®^ dsDNA BR Assay Kit (Thermo Scientific) and agarose gel electrophoresis. DNA was then forwarded to Otogenetics or Beijing Genomics Institute (BGI) for additional in‐house quality control assessments prior to WES.

For WES at Otogenetics, 3 μg of genomic DNA (gDNA) was sheared to yield 100–450 bp fragments. In‐solution whole‐exome capture and massively parallel sequencing was performed using the Agilent SureSelect^XT^ All Exon Kit 51 Mb. Enriched DNA fragments were sequenced on Illumina's HiSeq 2500 platform as paired‐end 100–125 base‐pair reads. On average, over 95% of exons were covered at >20×. The percentage of exome coverage was based on exons targeted by the 51 Mb All Exon v4 Kit which incorporates Consensus Coding Sequence (CCDS), NCBI Reference Sequence (RefSeq) and GENCODE annotations.

For WES at BGI, the gDNA samples were fragmented by Covaris, and, after two rounds of bead purification, the resulting gDNA fragments were mainly distributed between 200 and 400 bp. Then, AdA 5′‐ and 3′‐adaptors were ligated to the 5′‐ and 3′‐ends of the fragments, respectively. The AdA adaptor‐ligated fragments were amplified by PCR, and the PCR products were used for exon capture. A 58.95 Mb region was targeted for capture. The captured exon fragments were purified by DynabeadsM‐280 streptavidin bead purification and were further amplified by another round of PCR. Then, the PCR products were circularized and the resulting double strand (ds) circles digested with *Ecop15*. Among these digested fragments, small fragments were collected after bead purification. Similar to the AdA adaptor ligation, AdB adapters were ligated to both ends of the purified fragments and the fragments were then used for single strand (ss) circularization. The resulting ss circles were the final library products used on the CG Black Bird sequencing platform. Finally, high‐throughput sequencing was performed for each captured library.

### 2.4 Read mapping

Sequence reads (FASTQ) from Illumina (Otogenetics) were mapped to the human reference genome (NCBI build 37.1) with NextGENe^®^ (SoftGenetics). Using the consolidation and elongation functions of NextGENe, instrument sequencing errors were reduced and sequence reads were lengthened prior to variant analysis. The condensation tool polished the data for adequate coverage by clustering similar reads with a unique anchor sequence. Using this process, short reads were lengthened and reads with errors were filtered or corrected. To maximize the probability of detecting causal variants, all base changes occurring in ≥4 reads in any individual sample were classified as variants for downstream analyses. An Overall Mutation score of 5 was used as a cut‐off to filter read errors and reduce the effects of allelic imbalances. The Overall Mutation score is generated via a proprietary algorithm (SoftGenetics) to provide an empirical estimation of the likelihood that a given variant call is genuine and not an artifact of sequencing or alignment errors. This score is based on the concept of Phred scores, where quality scores are logarithmically linked to error probabilities. With NextGENe^®^ software, intergenic and deep intronic (≥12 nt from splice sites) variants were eliminated prior to downstream *in silico* analyses.

Complete Genomics (BGI) developed high‐speed mapping software capable of aligning read data to reference sequences. Using GRCh37 as the reference, the mapping is tolerant of small variations from a reference sequence, such as those caused by individual genomic variation, read errors, or unread bases. To support assembly of larger variations, including large‐scale structural changes or regions of dense variation, each arm of a DNA Nanoball (DNB) is mapped separately, with mate pairing constraints applied after alignment. Initially, mapping reads to the human reference genome is a constrained process that does not allow for insertions and deletions. All mate‐pair constraint‐satisfying paired‐end mappings are used to detect small variants. DNBs are then filtered and individual reads are optimized. Optimization collects reads likely to lie in regions of interest, using mate alignment information and performs local *de novo* assemblies.

### 2.5 Single‐nucleotide variants (SNVs) and small insertions and deletions (INDELS)

First, a list of shared variants was generated for pedigrees with two or more affected subjects analyzed with WES. For Otogenetics Illumina data, we eliminated SNVs and INDELS with minor allele frequencies (MAFs) ≥0.001 in the Exome Aggregation Consortium (ExAC; Lek et al., 2016) database or 1000 Genomes (1KG), variants with unbalanced reads (variant allele < 25%), and regions covered by <5 reads. For BGI data, we eliminated SNVs and INDELs with MAFs ≥0.001 in 1 KG or Exome Variant Server (EVS). Of note, both BGI and Otogenetics outputs contain inverted major/minor allele classifications for a subset of sequence variants (minor allele: MAF <0.001 or >0.999). All nonsynonymous SNVs were analyzed with dbNSFP (versions 3.3 to 3.5; Liu et al., 2016), CADD (Kircher et al., 2014) and REVEL (Ioannidis et al., 2016). Nonsynonymous SNVs with MetaLR (Dong et al., 2015) ranking scores >0.75, CADD phred scores >15, or REVEL scores >0.5 were retained for further evaluation. Nonsense SNVs, frameshift variants, synonymous SNVs, splice site SNVs, and other SNVs and INDELS (3′ and 5′ untranslated region [UTR] variants, downstream variants, intronic variants, noncoding variants and upstream variants) were analyzed with CADD +/− MutationTaster2 (Schwarz, Rodelsperger, Schuelke, & Seelow, 2010). Nonsense SNVs, frameshift variants, synonymous SNVs, splice site SNVs, other SNVs and INDELs with CADD_phred scores >15 were retained for further evaluation. All splice‐site SNVs were analyzed with dbscSNV1.1 (Jian et al., 2014), which contains precomputed ensemble scores, Ada and RF, for all potential splice‐site SNVs computed using AdaBoost and random forests, respectively. Splice‐site SNVs with Ada scores >0.6 or RF scores >0.6 were retained for further evaluation. Particular attention was paid to variants within the DYT13 (1p36.32‐p36.13; Bentivoglio et al., 1997; Valente et al., 2001) and DYT21 (2q14.3‐q21.3) loci. The DYT13 locus was identified via linkage analysis of a large 3‐generation pedigree with craniocervical and other anatomical distributions of dystonia. Similarly, the DYT21 locus was defined through linkage analysis of a Swedish kindred with apparently autosomal dominant inheritance of dystonia which included BSP is several affected subjects (Forsgren, Holmgren, Almay, & Drugge, 1988; Norgren, Mattson, Forsgren, & Holmberg, 2011). Detailed methods for analysis of BGI and Otogenetics information can be found in the Data S1.

REVEL, MetaLR and CADD scores were used to prioritize nonsynonymous missense variants for additional scrutiny whereas CADD and ExAC Probability of Loss‐of‐Function (LoF) intolerance (pLI) scores were used to prioritize nonsense SNVs and frameshift INDELs. MutationTaster was also used for analysis of small INDELs which are not scored by REVEL or MetaLR. Each category of variant (nonsynonymous, synonymous, splice‐site, nonsense, frameshift, other INDELs, and other SNVs) was ranked by *in silico* scores of deleteriousness. Population frequencies for the highest scoring variants were additionally assessed with genome Aggregation Database (gnomAD), NHLBI Exome Sequencing Project (ESP) Exome Variant Server (EVS) with particular attention to racial subcategories. All NCBI databases were queried with gene symbols and the names of encoded proteins. Particular attention was paid to data contained in PubMed, ClinVar, OMIM, and BioSystems. OMIM was searched for allelic disorders/phenotypes. MARRVEL and its link outs were used to explore available data related to animal models of homologs, genomic structural variants (DGV and DECIPHER), gene expression (GTex), and protein expression (ProteinAtlas). Gene expression was also analyzed with Allen Brain Atlas and BioGPS. Candidate genes were eliminated if not expressed in at least one “motor” region of the brain (striatum, cerebellum or frontal motor cortex). UniProt was used to access protein–protein interactions, sites of known or predicted posttranslational modifications and known or putative protein functions. Multiple sequence alignments were performed with Clustal Omega. A subset of candidate pathogenic variants was confirmed with bidirectional Sanger sequencing to exclude next generation sequencing read errors. After Sanger confirmation, cosegregation was assessed in individual pedigrees.

### 2.6 Copy number variant analysis

CNVkit (Talevich, Shain, Botton, & Bastian, 2016), a Python library and command‐line software toolkit to infer and visualize copy number variants (CNVs) from targeted DNA sequencing data, was used to detect CNVs in WES data generated by Otogenetics on the Illumina platform. CNVkit was designed for use on hybrid capture sequencing data where off‐target reads are present and can be used to improve copy number estimates. CNVkit normalizes read counts to a pooled reference and corrects for three main sources of bias: GC content, target footprint size, and repetitive sequences. For this purpose, Otogenetics provided us with WES data from 15 random subjects of unknown race and unknown geographic region of origin sequenced as part of unrelated projects using the Agilent SureSelect^XT^ All Exon Kit 51 Mb for exome capture and sequenced on Illumina's HiSeq 2500 platform.

CNVkit reports log2 copy ratios. Assuming pure samples and germline mutations, the log2 ratio should be −1.0 for a deletion mutation and infinity if both alleles are deleted. The log2 ratio is 0.585 for duplications and 1.0 for triplications. The relationship between the estimated copy number and the true copy number depends on a number of factors including read depth and number of probes covering a region of interest.

### 2.7 Sanger sequencing

PCR was performed using 40 ng of peripheral blood gDNA along with 200 nmol/L of each primer (Table S1) in a 10‐μl reaction volume with HotStarTaq^®^ Plus DNA polymerase from Qiagen. The following cycling conditions were employed: 95°C for 15 min; 35 cycles at 95°C for 10 s, 58°C for 30 s, and 72°C for 30 s.

### 2.8 PCR validation of copy number variants

Quantitative PCR (qPCR) was used for initial assessment of a random selection of predicted CNVs identified with CNVkit. Primers and probes for qPCR were designed with Roche's Universal Probe Library to cover (Table S1). qPCR was performed using 20 ng of template DNA and 200 nmol/L of each primer in a 10‐μl reaction volume with the LightCycler™ 480 system and Universal Taqman^®^ probes (Roche). The following cycling conditions were employed: 95°C for 5 min; 45 cycles at 95°C for 10 s, 58°C for 30 s, and 72°C for 12 s. Copy numbers were calculated against an endogenous control, *HLCS*, holocarboxylase synthetase. All assays were carried out in triplicate and means were used for calculating fold changes.

Digital PCR (dPCR) was then used for confirmation of select deletion and duplication CNVs identified with CNVkit. Literature mining as described for SNVs and small lNDELs was used to select genes with deletion log2 scores of −0.75 to −1.25 and covered by ≥4 probes, or genes with duplication log2 scores of 0.385 to 0.835 and covered by ≥4 probes. Primers and probes (FAM dye‐labeled) were designed via Roche's Universal Probe Library to encompass the estimated deletion regions (Table S1). The TaqMan copy number reference assay (Applied Biosystems 4403326) contained RNase P‐specific forward and reverse primers and VIC dye‐labeled TAMRA hydrolysis probe. RNase P, a single copy gene, is used as the reference for this work (Qin, Jones, & Ramakrishnan, 2008).

Reaction mixtures (4.0 μl) containing TaqMan gene‐expression master mix (Life Technologies), 20X GE sample loading reagent (Fluidigm 85000746), 20X gene‐specific assays, 20X TaqMan copy number reference assay (Applied Biosystems) and 1.2 μl target gDNA (20 ng/μl) was pipetted into each loading inlet of a 48.770 dPCR array (Fluidigm). The BioMark IFC controller MX (Fluidigm) was used to uniformly partition the reaction from the loading inlet into the 770 × 0.84 nl chambers and dPCR was performed with the Fluidigm BioMark System for Genetic Analysis. The Fluidigm dPCR software was used to count gene copy numbers. The quality thresholds were manually set specific to each assay, but consistent across all panels of the same assay. The CNV calculation is based on “relative copy number” so that apparent differences in gene copy numbers in different samples are not distorted by differences in sample amounts. The relative copy number of a gene (per genome) is expressed as the ratio of the copy number of a target gene to the copy number of a single copy reference gene in the sample. By using assays for the two genes (the gene of interest and the reference gene) with two fluorescent dyes on the same Digital Array IFC, we are able to simultaneously quantitate both genes in the same DNA sample. The ratio of these two genes is the relative copy number of the gene of interest.

### 2.9 Data availability

Primers (Table S1), WES variants examined with Sanger sequencing (Table S2), and potential CNVs examined with qPCR (Table S3) are included in Data S1. Comprehensive WES variant analysis for each pedigree is included in individual Excel workbooks (10012, 10014, 10035, 10036, 10043, 10064, 10076, 10178, 10193, 10274, 10455, 10908, 25056, 25069, 25215, 45263, 85020, NB0362_BGI, NG0369, NG0450, and NG1072_BGI).

## 3 RESULTS

### 3.1 BSP and BSP+ pedigrees

**Figure 1:**
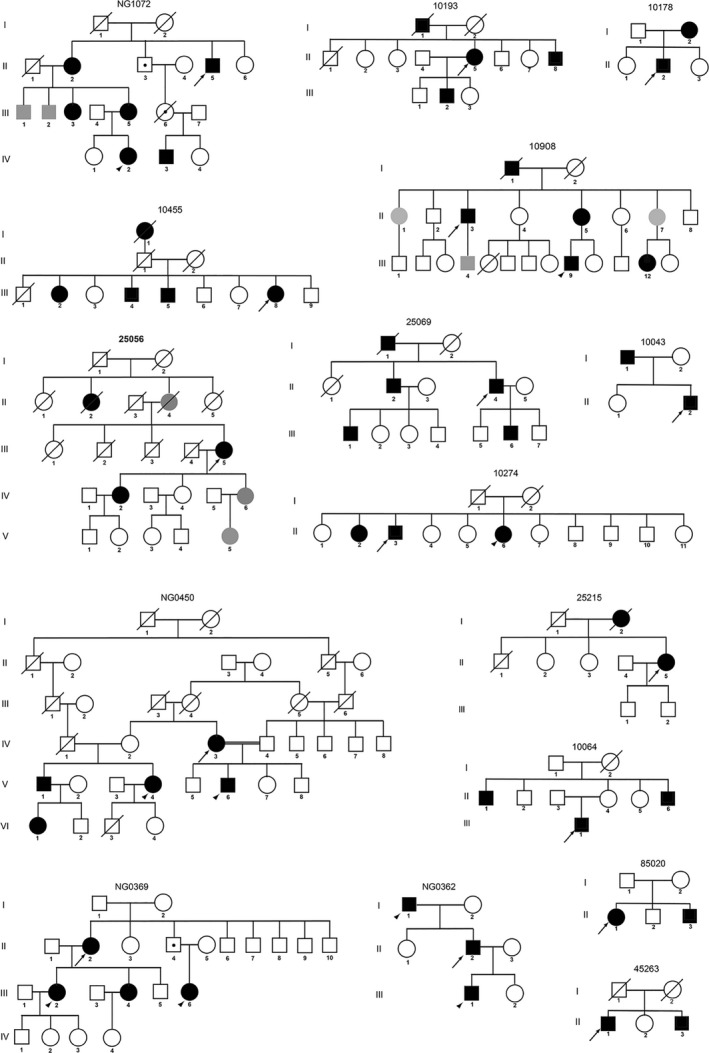
Blepharospasm (BSP) and BSP+ Pedigrees. Pedigrees with two or more affected individuals. Arrows, probands. Arrowheads, other family members analyzed with whole‐exome sequencing. White symbol, unaffected. Black symbols, BSP, BSP+ or other anatomical distribution of dystonia. Gray symbols, possibly affected

**Figure 2:**
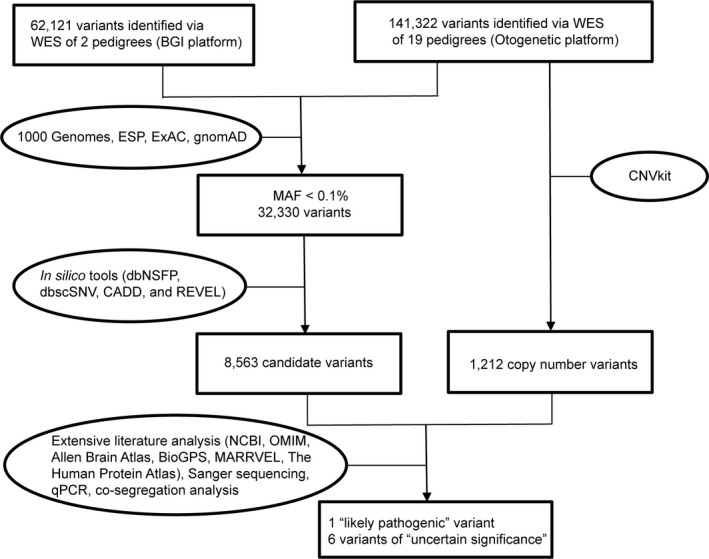
Flow chart for whole‐exome sequencing data analysis. Analysis of exomes sequenced by Beijing Genomics Institute (BGI) and Otogenetics. Otogenetics (Illumina) reads were mapped in house. BGI did not provide raw read data

Whole‐exome sequencing was completed on 31 subjects from 21 distinct pedigrees with either concordant or discordant BSP and BSP+ phenotypes (Table 1, Figures 1 and 2, Data S1). Exome coverage is provided in Tables 2 and 3. Depth of coverage was ≥10× and ≥20× for over 97.5% and 95% of the 31 exomes. Numbers of total and filtered variants are provided in Table 4.

**Table 2:** Exome coverage otogenetics (Illumina)

Subjects	Exon coverage			Mapped reads	Reads in exons (% of mapped)
	≥10× average	≥20× average	≥50× average		
NG0369‐II‐2	182,985 (98.71%)	180,451 (97.20%)	158,686 (85.48%)	40,359,835	27,882,382 (69.08%)
NG0369‐III‐2	183,285 (98.73%)	181,151 (97.58%)	164,561 (88.64%)	46,282,001	31,819,162 (68.75%)
NG0369‐III‐6	183,245 (98.71%)	181,036 (97.52%)	163,455 (88.05%)	45,818,817	31,595,919 (68.95%)
NG0450‐V‐4	183,339 (98.76%)	181,430 (97.73%)	167,149 (90.04%)	48,910,931	33,455,111 (68.41%)
NG0450‐V‐6	183,262 (98.72%)	180,540 (97.25%)	160,781 (86.61%)	44,674,009	30,667,372 (68.64%)
NG0450‐IV‐3	182,910 (98.53%)	180,235 (97.09%)	157,051 (84.60%)	38,518,463	26,893,126 (69.81%)
10012	183,177 (98.67%)	179,968 (96.94%)	151,762 (81.75%)	43,360,914	30,295,318 (69.86%)
10014	183,345 (98.76%)	180,745 (97.36%)	157,291 (84.73%)	45,133,245	30,866,426 (68.38%)
10035	183,449 (98.82%)	181,074 (97.54%)	159,805 (86.08%)	47,593,537	32,451,839 (68.18%)
10036	183,377 (98.78%)	180,492 (97.22%)	155,013 (83.50%)	43,455,430	29,831,744 (68.64%)
10043‐II‐2	182,658 (98.39%)	179,067 (96.46%)	149,939 (80.77%)	36,183,050	23,982,731 (66.28%)
10064	181,329 (97.67%)	174,416 (93.95%)	135,925 (73.22%)	31,906,497	23,887,178 (74.87%)
10076	181,156 (97.58%)	175,038 (94.29%)	137,975 (74.32%)	30,495,728	22,423,886 (73.53%)
10043‐I‐1	183,249 (98.71%)	181,131 (97.57%)	166,235 (89.54%)	50,010,351	34,444,302 (68.87%)
10178	183,260 (98.72%)	180,253 (97.10%)	157,001 (84.57%)	44,071,238	29,754,633 (67.51%)
10193	182,958 (98.55%)	179,944 (96.93%)	154,912 (83.44%)	40,787,072	27,819,791 (68.20%)
10274‐II‐3	183,257 (98.72%)	180,866 (97.43%)	162,983 (87.79%)	44,609,530	29,931,870 (67.09%)
10274‐II‐6	183,149 (98.66%)	181,247 (97.63%)	167,458 (90.2%)	54,207,882	36,192,334 (66.76%)
10455	183,030 (98.59%)	180,044 (96.98%)	156,811 (84.47%)	48,944,558	30,884,770 (63.10%)
10908‐II‐3	183,169 (98.67%)	181,017 (97.51%)	164,543 (88.63%)	47,084,143	32,159,148 (68.30%)
10908‐III‐9	183,065 (98.6%)	183,065 (97.35%)	163,351 (87.99%)	45,541,395	30,924,196 (67.90%)
25056	183,204 (98.68%)	180,273 (97.11%)	153,853 (82.87%)	45,212,675	30,858,562 (68.25%)
25069	182,022 (98.05%)	176,604 (95.13%)	142,875 (76.96%)	33,328,570	24,217,999 (72.66%)
25215	182,687 (98.41%)	179,180 (96.52%)	150,346 (80.99%)	37,771,677	25,469,621 (67.47%)
45263	183,442 (98.81%)	181,182 (97.61%)	163,127 (87.87%)	48,922,580	33,055,765 (67.56%)
85020	183,190 (98.68%)	180,786 (97.38%)	163,888 (88.28%)	58,478,165	35,599,118 (60.87%)

**Table 3:** Exome coverage BGI (Complete Genomics)

Subject	Bases on targets	Targets covered ≥ 1×	Targets covered ≥ 5×	Targets covered ≥ 10×	Targets covered ≥ 20×
NG0362‐III‐1	58,970,115	99.56%	98.68%	97.64%	95.28%
NG1072‐II‐5	58,970,115	99.57%	98.72%	97.70%	95.36%
NG0362‐II‐2	58,970,115	99.56%	98.66%	97.60%	95.25%
NG0362‐I‐1	58,970,115	99.59%	98.76%	97.73%	95.32%
NG1072‐IV‐2	58,863,950	99.54%	98.66%	97.62%	95.27%

**Table 4:** Total and filtered variants

Pedigree (# subjects)	# common variants (SNVs + INDELs)	Potentially pathogenic variants							Platform
		Nonsynonymous SNVs	Nonsense SNVs	Synonymous SNVs	Splice site SNVs	Frame‐ shift	Other SNVs & indels	CNVs	
NG0362 (3)	30,704	68	4	9	5	7	32	NA	Complete Genomics
NG1072 (2)	31,417	63	1	8	4	5	42	NA	Complete Genomics
NG0369 (3)	3,771	60	2	8	2	14	232	217	Illumina
NG0450 (3)	3,749	48	3	8	0	13	214	145	Illumina
10043 (2)	4,233	82	3	10	1	20	184	46	Illumina
10274 (2)	5,462	141	6	10	2	25	26	110	Illumina
10908 (2)	4,665	79	2	9	0	19	227	46	Illumina
10012 (1)	6,511	118	3	9	1	6	243	60	Illumina
10014 (1)	7,255	173	7	21	2	29	272	69	Illumina
10035 (1)	7,016	141	7	16	4	23	251	38	Illumina
10036 (1)	6,954	137	3	4	2	19	234	41	Illumina
10064 (1)	14,196	258	5	36	9	30	347	50	Illumina
10076 (1)	14,357	178	9	29	1	17	340	29	Illumina
10178 (1)	7,865	127	8	21	2	33	239	14	Illumina
10193 (1)	7,136	129	7	20	1	22	213	42	Illumina
10455 (1)	7,551	167	7	24	1	25	262	80	Illumina
25056 (1)	7,196	170	6	16	4	23	254	61	Illumina
25069 (1)	9,064	145	4	22	3	23	254	11	Illumina
25215 (1)	7,017	176	5	19	5	23	256	52	Illumina
45263 (1)	9,340	139	2	19	3	25	277	25	Illumina
85020 (1)	7,984	151	3	22	4	31	246	77	Illumina

SNVs, single nucleotide variants; INDELs, small deletion and insertions; CNVs, copy number variants; NA, not available.

SNVs and INDELs with (MAFs) >0.001 (1 KG or EVS for Complete Genomics/BGI; and ExAC for Illumina/Otogenetics). Nonsynonymous SNVs: CADD phred score >15 or MetaLR >0.75 or REVEAL >0.5. Nonsense SNVs: CADD phred score >15. Synonymous SNVs: CADD phred score >15. Splice‐site SNVs: CADD phred score >15 or ada_score >0.6 or rf_score >0.6. Frame shift: CADD phred score >15. Other SNVs & INDELs: CADD phred score >15.

CNVs: all generated via analysis with CNVkit.

### 3.2 *CACNA1A* INDEL in a three‐generation pedigree with BSP

**Figure 3:**
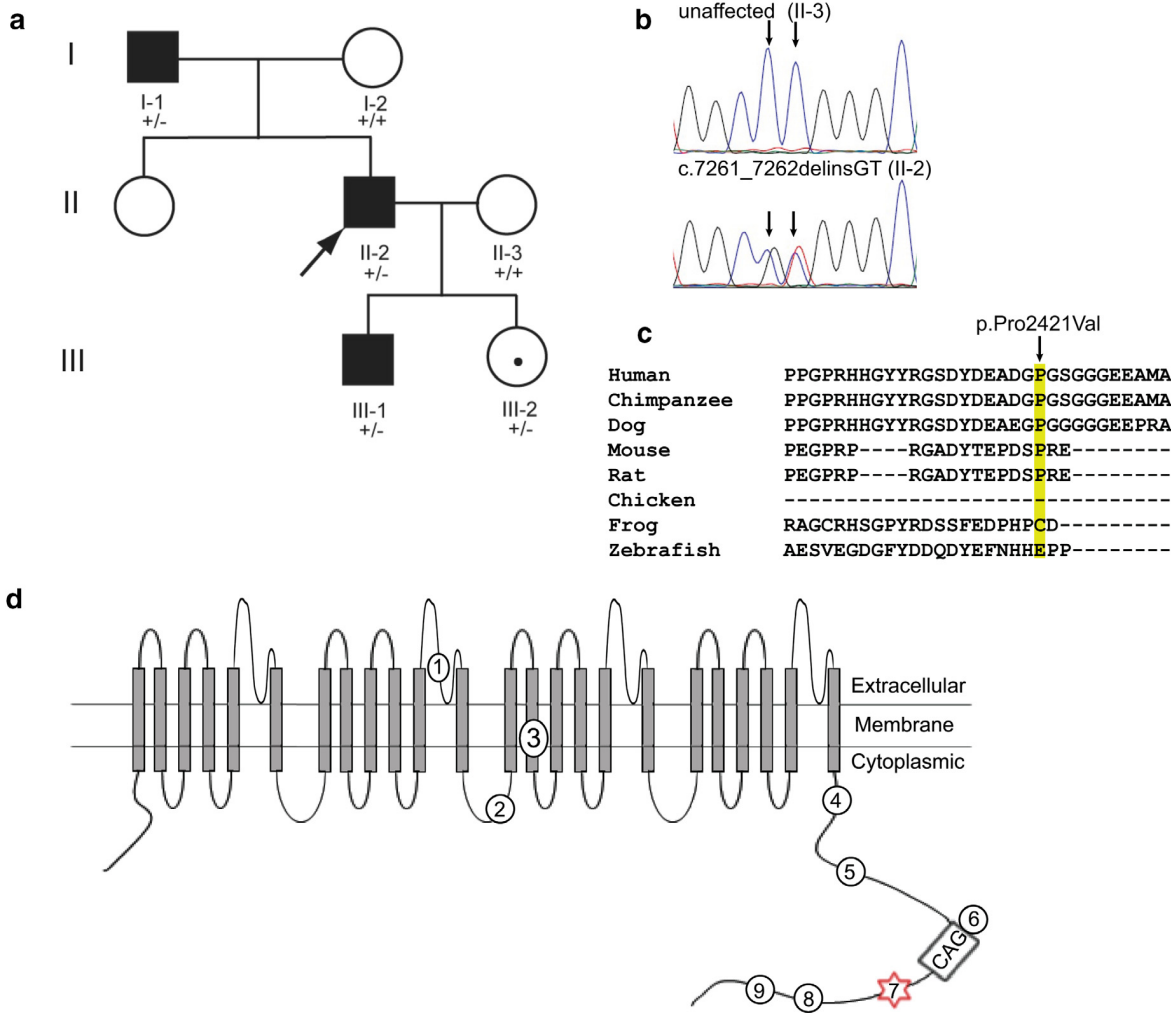
*CACNA1A* INDEL Identified in a Multigenerational Pedigree with BSP. (a) Family NG0362 with BSP. Three affected (I‐1, II‐2 and III‐1) individuals were selected for WES. +/+, wild‐type; +/‐, heterozygous for *CACNA1A* c.7261_7262delinsGT. (b) Electropherograms of unaffected family member (II‐3) and subject with BSP (II‐2). (c) Multiple sequence alignment shows evolutionary conservation of Pro2421 among mammals. (d) Location of disease‐associated variants in the α‐1A subunit of P/Q type, voltage‐dependent, calcium channels: (1) Thr666Met variant linked to familial hemiplegic migraine and early‐onset cerebellar atrophy (Naik et al., 2011; Ophoff et al., 1996), (2) variant (c.3772delC) predicted to cause a frameshift and truncated protein or, more likely, nonsense‐mediated decay in a man with interictal BSP and episodic ataxia type 2 (Spacey et al., 2005), (3), splice‐site variant associated with episodic ataxia type 2 (Ophoff et al., 1996), (4) Ile1811Leu variant associated with familial hemiplegic migraine (Ophoff et al., 1996), (5), Glu2080Lys variant linked to sporadic hemiplegic migraine (Thomsen et al., 2008), (6), CAG expansion associated with spinocerebellar ataxia type 6 (SCA6) and dystonia (Kuo et al., 2017; Sethi & Jankovic, 2002; Zhuchenko et al., 1997), (7) Pro2421Val variant associated with BSP in our multigenerational pedigree, (8), Pro2479Leu associated with sporadic hemiplegic migraine (Thomsen et al., 2008), and (9) His2481Gln associated with sporadic hemiplegic migraine (Thomsen et al., 2008)

A novel *CACNA1A* INDEL (c.7261_7262delinsGT [NM_001127222.1], p.Pro2421Val [NP_001120694.1]) was identified in three males and one asymptomatic female family member from a three‐generation pedigree with BSP (Figure 3, Tables 1, 5, 8 and S2; Data S1). Complete Genomics outputted this variant as two contiguous SNVs. This INDEL is not reported in control databases (ExAC, 1KG or gnomAD) and predicted to be deleterious by CADD (Phred score = 19.51) and MutationTaster (disease causing, probability value: 1.0). However, two contiguous SNVs are reported in gnomAD (19:13318386 and 19:13318387) with very similar allele frequencies (211/118674 and 207/119456). Analysis of read data suggests that the majority of these SNVs are, in fact, part of the c.7261‐7262delinsGT INDEL. The 19:13318386G/A variant is present at relatively high frequency in the Finnish population (1.49E‐02) with a much lower allele frequency of (6.76E‐04) in non‐Finnish Europeans and quite rare in other racial populations. The identified amino acid substitution is located in the C‐terminal, intracellular domain of the encoded voltage‐dependent P/Q‐type calcium channel subunit α‐1A, which is conserved among mammals (Figure 3). We did not screen other variants for cosegregation given previously established associations between *CACNA1A* and dystonia. Five SNVs had CADD_phred scores >15 and REVEL scores >0.5 but none had a MetaLR score >0.75, REVEL score >0.75 and CADD_phred score >30. A frameshift INDEL in *MMP28* with a CADD_phred score of 34 is reported in ExAC and gnomAD. Four nonsense SNVs had CADD_phred scores >30 but two are reported in ExAC and gnomAD and none seem biologically plausible candidates.

**Table 5:** BSP‐associated sequence variants identified with whole‐exome sequencing, *in silico* analyses, and cosegregation analyses

Pedigree	Phenotype	Gene	cDNA/Accession number	Protein	ExAC	gnomAD	dbSNP	MutationTaster2	CADD	MetaLR	REVEL
10908	BSP+/BSP	*REEP4*	c.109C>T (NM_025232.3)	p.Arg37Trp	1.66E‐05 (2/120748)	2.03E‐05 (5/246118)	rs780399718	Disease causing	34.0	0.960	0.767
NG0362	BSP	*CACNA1A*	c.7261_7262delinsGT (NM_001127222.1)	p.Pro2421Val	NA	NA	NA	Disease causing	19.5	NA	NA
NG0369	BSP	*TOR2A*	c.568C>T (NM_130459.3)	p.Arg190Cys	5.84E‐05 (7/119868)	4.07E‐05 (10/245852)	rs376074923	Disease causing	34.0	0.811	0.548
NG1072	BSP	*ATP2A3*	c.1966C>T (NM_005173.3)	p.Arg656Cys	5.51E‐04 (66/119706)	6.63E‐04 (183/276114)	rs140404080	Disease causing	34.0	0.992	0.872
10043	BSP+	*GNA14*	c.989_990delCA (NM_004297.3)	p.Thr330ArgfsTer67	1.65E‐05 (2/121284)	1.23E‐05 (3/244472)	NA	disease causing	36.0	NA	NA
10043	BSP+	*HS1BP3*	c.94G>T (NM_022460.3)	p.Gly32Cys	NA	NA	NA	Disease causing	34.0	0.803	0.454

NA, not available.

### 3.3 *REEP4* missense variant

**Figure 4:**
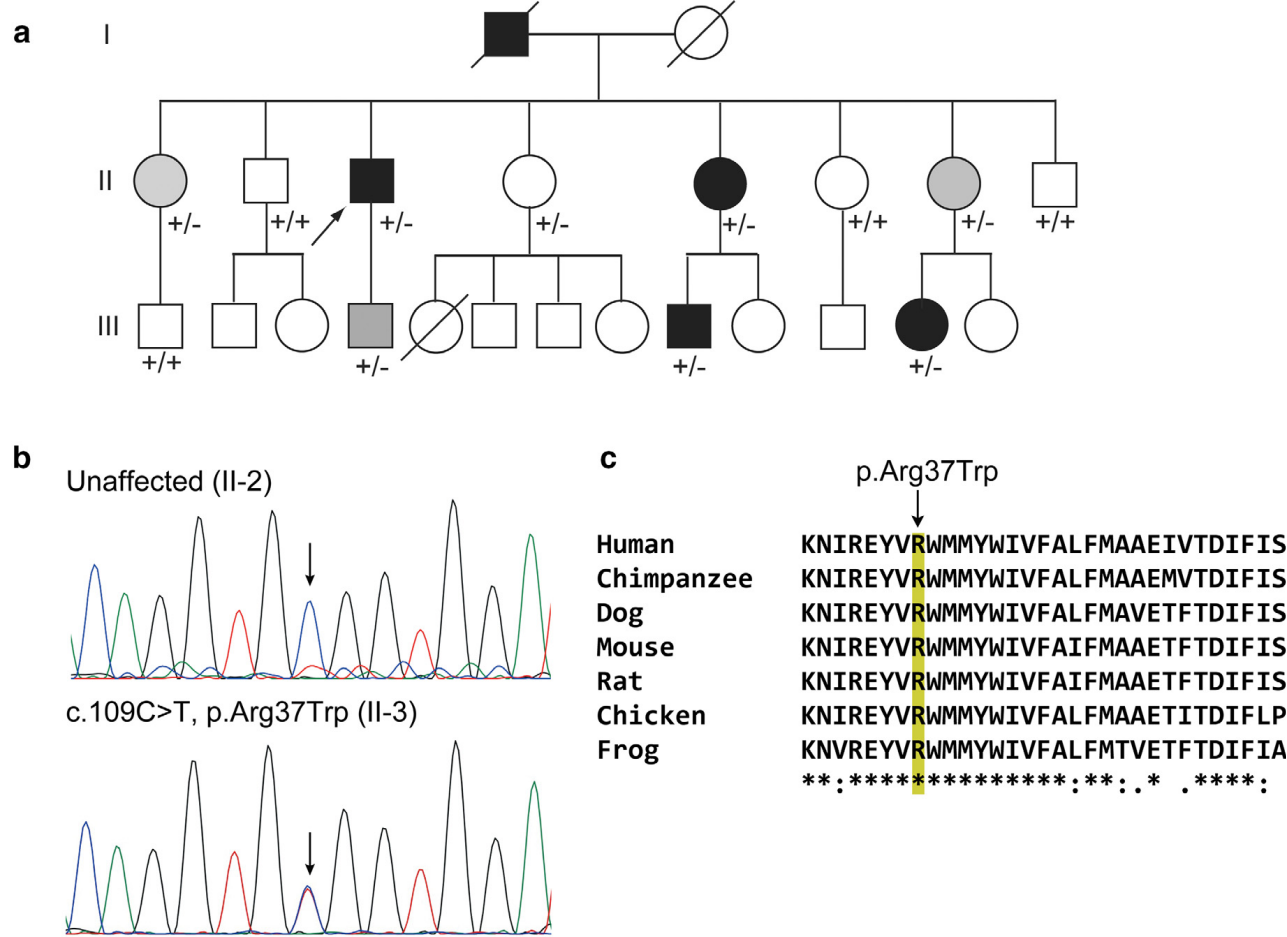
*REEP4* Variant in African–American Pedigree with BSP+ and BSP. (a) Family 10908 with BSP+ and BSP. Two affected (II‐3 and III‐9) individuals were selected for WES. +/+, wild‐type; +/‐, heterozygous for *REEP4* c.109C>T. (b) Electropherograms of unaffected family member (II‐2) and subject with BSP+ (II‐3). (c) Multiple sequence alignment shows evolutionary conservation of Arg37 among vertebrates

A nonsynonymouse SNV in *REEP4* (c.109C>T [NM_025232.3], p.Arg37Trp [NP_079508.2]) was identified in seven subjects with BSP+ or BSP and one asymptomatic female family member from a three‐generation African–American pedigree (Figure 4, Tables 1, 5, 8 and S2; Data S1). This variant is present at very low frequency in gnomAD and predicted to be deleterious by *in silico* analysis, including CADD (phred score = 34), REVEL (0.767), MetaLR (0.960), and MutationTaster2 (disease causing, probability value: 1.0). In gnomAD, this variant is not present in 15,290 African alleles. The p.Arg37Trp variant alters an amino acid that is highly conserved among vertebrates as shown by the multiple pairwise alignments generated with Clustal Omega (Figure 4).

### 3.4 *TOR2A* missense variant

**Figure 5:**
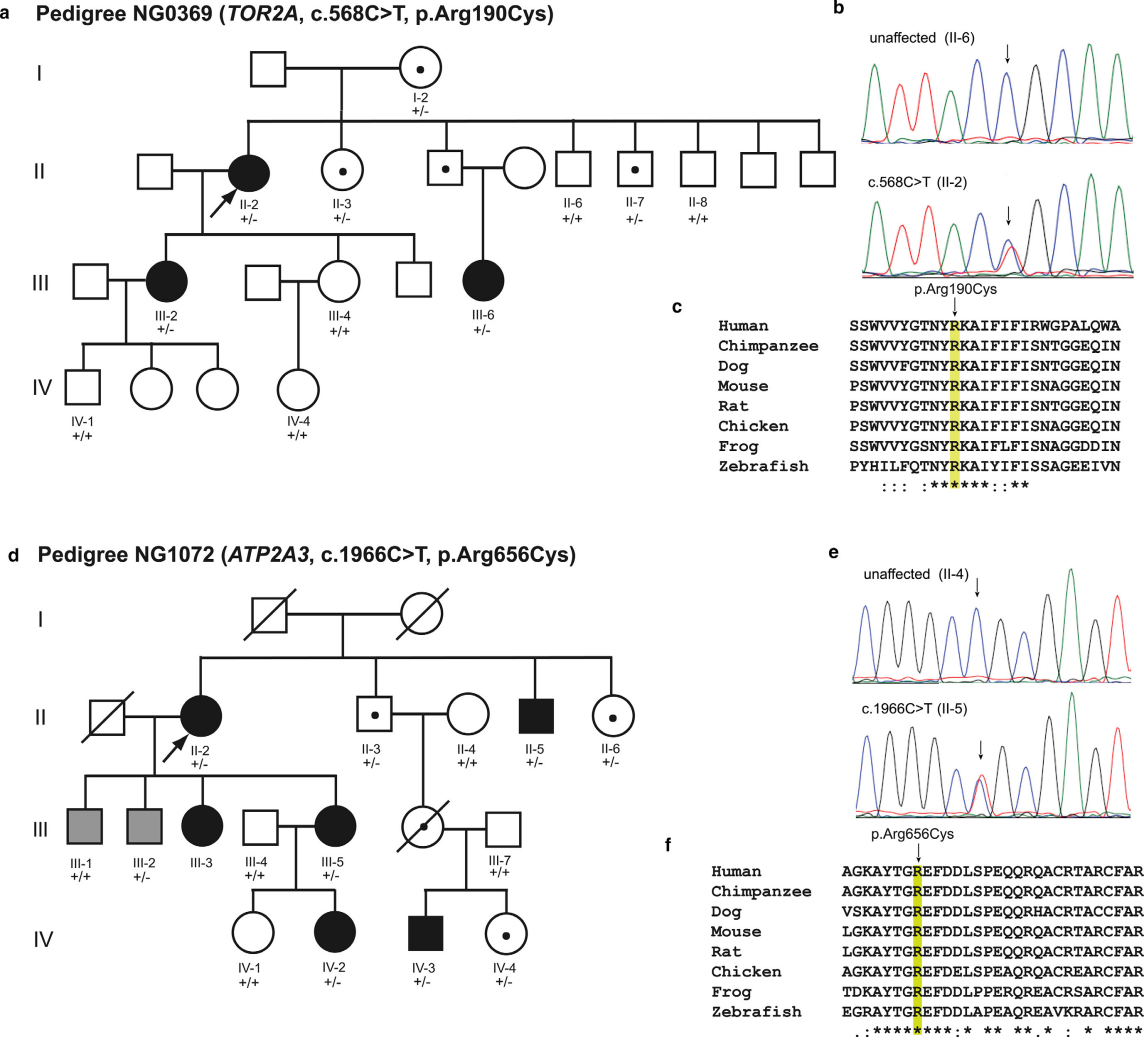
*TOR2A* and *ATP2A3* Variants in Multigenerational Pedigrees with BSP. (a) Family NG0369 with BSP. Three affected (II‐2, III‐2 and III‐6) individuals were selected for WES. +/+, wild‐type; +/−, heterozygous for *TOR2A* c.568C>T. (b) Electropherograms of unaffected family member (II‐6) and subject with BSP (II‐2). (c) Multiple sequence alignment shows evolutionary conservation of Arg190 among vertebrates. (d) Discordant pedigree NG1072 with BSP, cervical dystonia, and arm dystonia. Two affected individuals were selected for WES (II‐2, IV‐2). +/+, wild‐type; +/−, heterozygous for *ATP23* c.1966C>T. White symbol, unaffected. Black symbol, BSP, BSP+ or other anatomical distribution of dystonia. Gray symbol, possibly affected. (e) Electropherograms of unaffected family member (II‐4) and subject with BSP (II‐2). (f) Multiple sequence alignment shows evolutionary conservation of Arg656 among vertebrates

A *TOR2A* nonsynonymous SNV (c.568C>T [NM_130459.3], p.Arg190Cys [NP_569726.2]) was identified in three subjects with BSP and three asymptomatic members from a four generation pedigree (Figure 5; Tables 1, 5, 8 and S2; Data S1). This variant is present at low frequency in ExAC (5.84e‐05) and predicted to be deleterious by *in silico* analysis including CADD (phred score = 34), REVEL (0.548), MetaLR (0.811), and MutationTaster2 (disease causing, probability value: 1.0). The p.Arg190Cys variant alters an amino acid that is highly conserved among vertebrates as shown by the multiple pairwise alignments generated with Clustal Omega (Figure 5). *TOR2A* encodes torsin family 2 member, a known interactor with dystonia‐associated protein torsinA (BioGRID). Nonsense variants in *PCDH15* and *GTDC1* were also detected in all three affected subjects and have CADD_phred scores >30 but pLI scores of 0. *PCDH15* and *GTDC1* have 28 and 15 LoF variants in ExAC, respectively. *FRG1* variants detected with WES are likely due to mapping errors caused by related genomic sequences.

### 3.5 *ATP2A3* missense variant

An *ATP2A3* nonsynonymous SNV (c.1966C>T [NM_005173.3], p.Arg656Cys [NP_001120694.1]) was identified in five affected subjects, one possibly affected subject, and three asymptomatic members of discordant Family NG1072 (Figure 5; Tables 1, 5, 8 and S2; Data S1). Predicted to be highly deleterious by all *in silico* analysis (CADD_phred score = 34, REVEL score = 0.872, MetaLR = 0.99175, MutationTaster2 [disease causing, probability value: 1.0]), this variant (rs140404080) is reported in ExAC (5.51E‐04) and gnomAD (6.63E‐04) with a population frequency of approximately 0.1%. The Arg656Cys variant alters an amino acid that is highly conserved among vertebrates (Figure 5). Another candidate variant in *MYH13* (rs7807826) did not completely cosegregate with dystonia in this pedigree (Table S2, Data S1). Moreover, expression of *MYH13* is mainly restricted to the extrinsic eye muscles. A nonsense variant in *NOS2* (NM_000625.4: c.2059C>T, p.Arg687*; CADD_phred = 36) was shared by the two affected individuals analyzed with WES but *NOS2* is expressed at only low levels in brain and *Nos2* ^‐/‐^ mice have not been reported to manifest positive or negative motor signs. *ATP2A3* is highly expressed in cerebellar Purkinje cells (Allen Brain Atlas) and is a member of the P‐type ATPase superfamily that includes the gene (*ATP1A3*) causally associated with rapid‐onset dystonia‐Parkinsonism (DYT12).

### 3.6 *GNA14* and *HS1BP3* variants in pedigree with BSP+ and Parkinsonism

**Figure 6:**
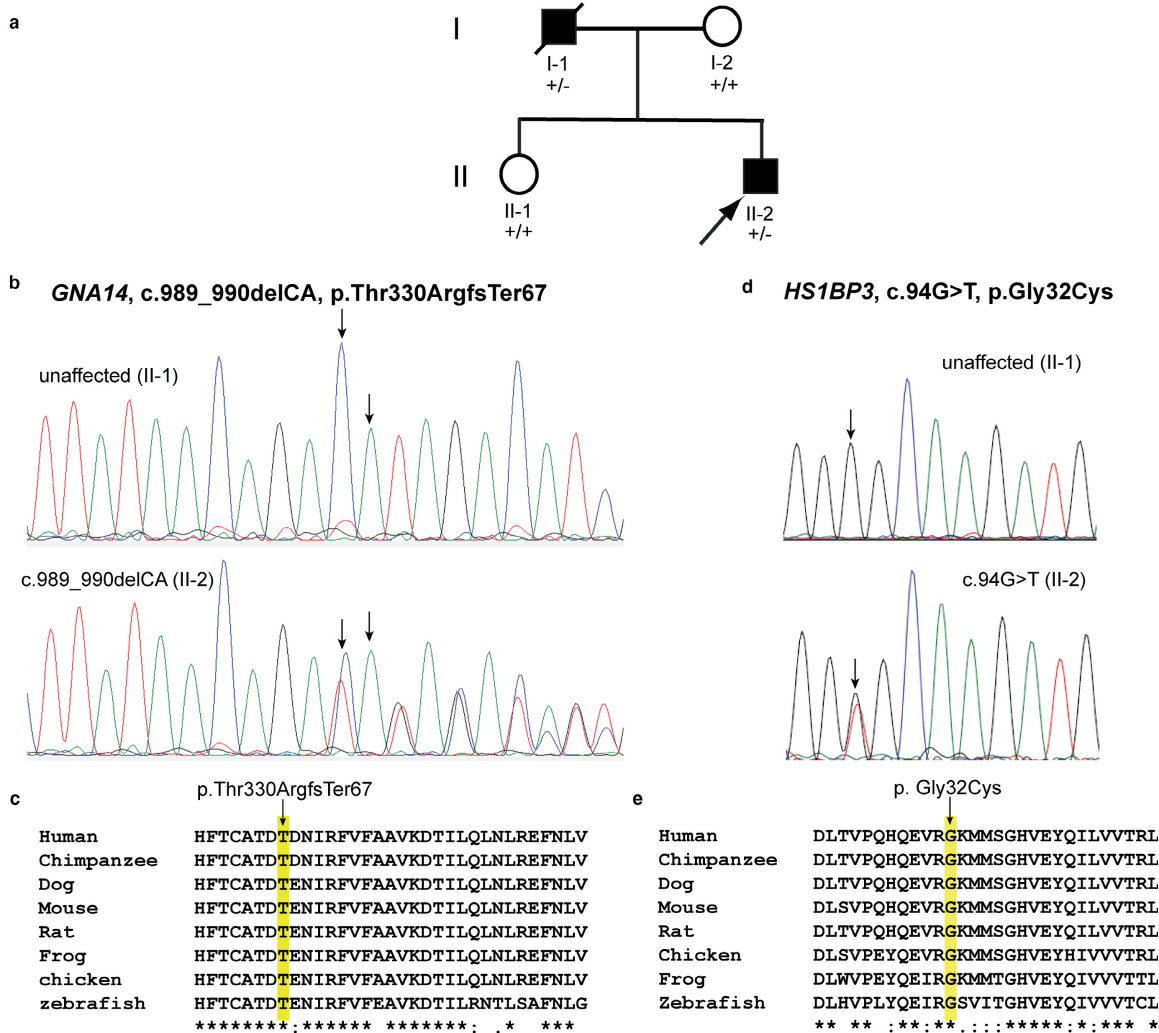
*GNA14* and *HS1BP3* Variants in Father and Son with BSP+. (a) Pedigree 10043. The proband has BSP+ and levodopa‐responsive Parkinsonism. His father had BSP+ and both were selected for WES. +/+, wild‐type; +/−, heterozygous for variants in *GNA14* and *HS1BP3*. (b) Electropherograms of unaffected family member (II‐1) and proband (II‐2) show *GNA14* variant. (c) Multiple sequence alignment shows evolutionary conservation of Thr330 among vertebrates. (d) Electropherograms of unaffected family member (II‐1) and proband (II‐2) show *HS1BP3* variant. (e) Multiple sequence alignment shows evolutionary conservation of Gly32 among vertebrates

A novel *HS1BP3* nonsynonymous SNV (c.94C>A [NM_022460.3], p.Gly32Cys [NP_071905.3]) was found in a father and son with severe BSP+ (Family 10043; Figure 6; Tables 1, 5, 8 and S2; Data S1). The deceased father had two brothers with clinical diagnoses of Parkinson disease (PD). The proband has BSP, mild lower facial dystonia, cervical dystonia and laryngeal respiratory dystonia. The laryngeal respiratory dystonia required treatment with a tracheostomy. The proband developed levodopa‐responsive Parkinsonism approximately 15 years after the onset of his dystonia. An ioflupane I‐123 dopamine transporter scan showed nigrostriatal denervation. The c.94C>A [NM_022460.3] variant is not reported in ExAC, 1KG or gnomAD, and is predicted to be deleterious by all *in silico* analysis (CADD_phred score = 34, REVEL = 0.454, MetaLR = 0.803). Of note, a different variant in *HS1BP3* (p.A265G) was previously associated with essential tremor (ET), a disorder potentially related to the adult‐onset dystonias through common genetics (Higgins et al., 2005). The p.Gly32Cys variant alters an amino acid that is highly conserved among vertebrates (Figure 6).

A *GNA14* frameshift variant (c.989_990del [NM_004297.3], p.Thr330ArgfsTer67 [NP_004288.1]) was also identified in the same pedigree (Family 10043) and is present at low frequency in gnomAD (1.23E‐05) (Figure 6; Tables 1, 5, and 8 and S2; Data S1). This *GNA14* variant is predicted to be deleterious by CADD (phred score = 36) and MutationTaster2 (disease causing, probability value: 1.0). *GNA14* encodes G protein subunit α14 which shows modest expression in brain, particularly the striatum and cerebellum (Human Protein Atlas). Recently, somatic mutations in *GNA14* have been linked to congenital and sporadic vascular tumors (Lim et al., 2016). Mutations in another G protein, Gα(olf), are associated with various anatomical distributions of mainly adult‐onset dystonia.

### 3.7 *DNAH17* variants found in pedigree and isolated subject with BSP

**Figure 7:**
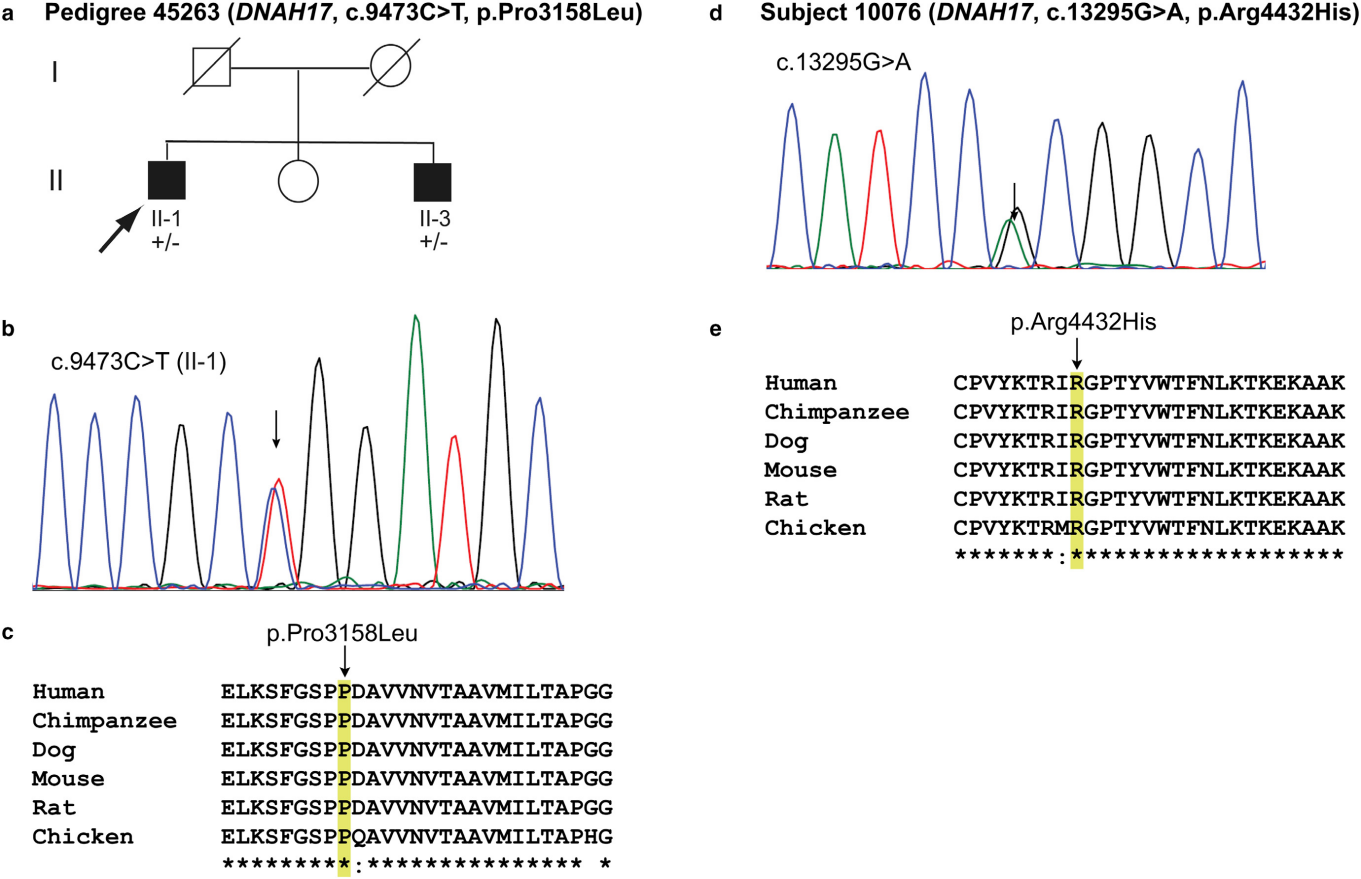
*DHAH17* Variants in Pedigree and Isolated Subject with BSP. (a) Pedigree 45263 with BSP. +/+, wild‐type; +/−, heterozygous for variant in *DNAH17*. (b) Electropherogram of proband (II‐2) showing *DNAH17* variant. (c) Multiple sequence alignment shows evolutionary conservation of Pro3158 among vertebrates. (d) Electropherogram of subject 10076 showing c.13295G>A variant. (e) Multiple sequence alignment shows evolutionary conservation of Arg4432 among vertebrates

Deleterious variants in *DNAH17* were identified in two brothers with BSP and one isolated case of BSP (Figure 7; Tables 1, 6, 8 and S2, Data S1). Both variants are present at low frequency in ExAC and gnomAD. *DNAH17* encodes dynein axonemal heavy chain 17. The FANTOM5 dataset reports expression of *DNAH17* in testes and brain (hippocampus, caudate and cerebellum; Kawaji, Kasukawa, Forrest, Carninci, & Hayashizaki, 2017). *DNAH17* has not yet been linked to any other neurological or non‐neurological disease. A roundworm homolog (*dhc‐1*) of human *DNAH17* is involved in cytokinesis, microtubule‐based movement, mitotic spindle organization, meiotic nuclear division and nervous system development (MARRVEL).

**Table 6:** Candidate genes common to two or more pedigrees

Gene	Pedigree	Variant (Accession Number)	ExAC	gnomAD	dbSNP	MutationTaster2	CADD	MetaLR	REVEL
*TRPV4*	10274	c.1337G>T p.Arg446Leu (NM_021625.4)	2.64E‐04 (32/121218)	2.93E‐04 (81/276794)	rs143502097	Disease causing	34.0	0.943	0.845
*TRPV4*	NG0450	c.745T>A p.Tyr249Asn (NM_001177431.1)	1.33E‐04 (16/120694)	1.01E‐04 (28/276982)	rs200210023	Disease causing	27.7	0.876	0.779
*TRPV4*	10035	c.769C>G p.Leu257Val (NM_021625.4)	8.04E‐04 (97/120672)	7.47E‐04 (207/276982)	rs56217500	Disease causing	23.8	0.958	0.669
*CAPN11*	10076	c.425T>C p.Leu142Pro (NM_007058.3)	NA	3.23E‐05 (1/30926)	rs111320370	Disease causing	32	0.982	0.918
*CAPN11*	10455	c.425T>C p.Leu142Pro (NM_007058.3)	NA	3.23E‐05 (1/30926)	rs111320370	Disease causing	32	0.982	0.918
*DNAH17*	10076	c.13295G>A p.Arg4432His (NM_173628.3)	6.60E‐05 (8/121400)	6.89E‐05 (19/275784)	rs775238626	Disease causing	35	0.763	0.477
*DNAH17*	45263	c.9473C>T p.Pro3158Leu (NM_173628.3)	9.93E‐05 (12/120872)	9.38E‐05 (26/277132)	rs371315860	Disease causing	25.3	0.947	0.613
*VPS13C*	10014	c.10954C>T p.Arg3652Ter (NM_020821.2)	1.84E‐04 (21/120740)	1.85E‐04 (50/270798)	rs138846118	Disease causing	49	NA	NA
*VPS13C*	10455	c.9605C>G p.Ala3202Gly (NM_020821.2)	8.45E‐06 (1/118378)	4.55E‐06 (1/219796)	rs750390167	Disease causing	33	0.869	0.598
*UNC13B*	25069	c.4192A>G p.Thr1398Ala (NM_006377.3)	NA	NA	NA	Disease causing	24	0.840	0.847
*UNC13B*	25215	c.4754G>A p.Arg1585His (NM_006377.3)	2.41E‐04 (29/120560)	2.56E‐04 (71/277062)	rs148652179	Disease causing	34	0.952	0.644
*SPTBN4*	10012	c.1594G>A p.Glu532Lys (NM_020971.2)	4.16E‐05 (5/120268)	6.16E‐05 (17/275852)	rs201278278	Disease causing	31	0.547	0.185
*SPTBN4*	10455	c.1543C>T p.Arg515Cys (NM_020971.2)	1.66E‐05 (2/120186)	4.48E‐05 (11/245642)	rs749869944	Disease causing	34	0.584	0.316
*MYOD1*	10178	c.485C>T p.Ala162Val (NM_002478.4)	2.97E‐04 (34/114390)	3.65E‐04 (95/260404)	rs150053079	Disease causing	23.1	0.977	0.678
*MYOD1*	10193	c.485C>T p.Ala162Val (NM_002478.4)	2.97E‐04 (34/114390)	3.65E‐04 (95/260404)	rs150053079	Disease causing	23.1	0.977	0.678
*MRPL15*	10036	c.485_498delTAGCTATTGCTGCC p.Leu162HisfsTer109 (NM_014175.3)	NA	NA	NA	Disease causing	35	NA	NA
*MRPL15*	10178	c.201delT p.Fhe67LeufsTer30 (NM_014175.3)	3.05E‐04 (37/121336)	3.93E‐04 (109/277238)	NA	Disease causing	26.8	NA	NA

ExAC, Exome Aggregation Consortium; CADD, Combined Annotation Dependent Depletion; REVEL, Rare Exome Variant Ensemble Learner; NA, not available.

### 3.8 Copy number variants

CNVkit called from 11 to 217 CNVs per shared exome. Assessing randomly selected CNVs with qPCR showed high discordancy (Table S3), particularly for variants that did not have log2 ratios near −1.0. We then focused on CNVs with log2 ratios compatible with a single‐copy gain (~0.585) or single‐copy loss (−1.0) using dPCR. Deletions in *LILRA3* were confirmed in three unrelated subjects with BSP (Table 7). *LILRA3* (OMIM 604818) deletions are common in the general population and may increase risk for HIV infection and some autoimmune disorders (Ahrenstorf et al., 2017; Du et al., 2015). A deletion in *BTNL3* (OMIM 606192) and duplications in *SLC2A14* (OMIM 611039), *SLC2A3* (OMIM 138170), *TOP3B* (OMIM 603582), and *UNK* (616375) were identified in single exomes (Tables 7 and 8). *UNK* is expressed at high levels in brain (Allen Brain Atlas, BioGPS, and The Human Protein Atlas) and plays an important role in the development of neuronal morphology. Two *UNK* duplications are reported in ExAC. To date, UNK has not been linked to any medical disorder (OMIM). Copy number analysis of *GOLGA8A* (Chr15) was compromised by the presence of pseudogenes and a homolog with very close sequence similarity on Chr15.

**Table 7:** Confirmation of CNV variants using Digital PCR of Genomic DNA

Patient ID	Gene	hg19 CNV Coordinates	Log2Ratio	Digital PCR	
				Gene/RNASE P	CNV
10455	*BTNL3*	Chr5: 180416000–180429824	−0.95	0.50	Deletion
10036	*LILRA3*	Chr19: 54801997–54804319	−1.13	0.62	Deletion
10178	*LILRA3*	Chr19: 54801997–54804319	−0.91	0.55	Deletion
10193	*LILRA3*	Chr19: 54801997–54804319	−1.04	0.60	Deletion
25056	*SLC2A14*	Chr12: 7984292–8043706	0.53	1.52	Duplication
25056	*SLC2A3*	Chr12: 8074017–8088678	0.53	1.51	Duplication
25215	*TOP3B*	Chr22: 22312829–22330136	0.57	1.41	Duplication
85020	*UNK*	Chr17: 73808156–73820465	0.58	1.50	Duplication
25056	*CLEC18B*	Chr16: 74443499–74452124	−1.20	1.04	Normal
10036	*CYP2A7*	Chr19: 41381608–41386459	−1.08	1.00	Normal
10036	*LRRC49*	Chr15: 71229066–71305260	−0.92	1.05	Normal
10036	*RRP7A*	Chr22: 42908850–42912408	−0.97	1.01	Normal
25056	*GOLGA8A*	Chr15: 34673679–34681975	−0.93	1.45	Duplication
45263	*GOLGA8A*	Chr15: 34677244–34681975	−0.94	1.08	Normal
85020	*GOLGA8A*	Chr15: 34677244–34681975	−1.08	1.15	Normal

**Table 8:** Candidate gene literature mining

Gene	Protein	Function	ExAC pLI	ExAC Missense Z‐score	Diseases	Neural localization^a^
*CACNA1A*	Calcium channel, voltage‐dependent, P/Q type, alpha 1A subunit	Calcium ion transmembrane transport	1.00	7.23	SCA6, EA‐2, hemiplegic migraine, dystonia	High expression in cerebellum, especially in Purkinje cells
*REEP4*	Receptor accessory protein 4	Microtubule‐binding, endoplasmic reticulum and nuclear envelope protein	0.18	0.20	NA	Purkinje cells, cerebellar nuclear neurons
*TOR2A*	Torsin family 2, member A	ATP binding	0.06	0.04	NA	Moderate expression in brain
*ATP2A3*	ATPase, Ca++ transporting, ubiquitous	Calcium ion transport	0.06	3.13	NA	High expression in cerebellum, especially in Purkinje cells
*GNA14*	Guanine nucleotide‐ binding protein (G protein), alpha 14	Adenylate cyclase‐modulating G‐protein coupled receptor signaling pathway	0.00	−0.25	NA	Moderate expression in brain
*HS1BP3*	HCLS1‐binding protein 3	Regulation of apoptotic process	0.00	−0.24	Associated with familial essential tremor	Moderate expression in brain
*NEFH*	Neurofilament protein, heavy polypeptide	Axon development	0.00	0.88	Charcot‐Marie‐Tooth disease Type 2CC, sporadic amyotrophic lateral sclerosis	High expression in cerebellum, especially in Purkinje cells
*RWDD2A*	RWD domain‐ containing 2A	NA	0.00	0.64	NA	Moderate expression in brain
*TRPV4*	Transient receptor potential cation channel, subfamily V, member 4	Actin cytoskeleton reorganization, calcium ion transmembrane transport	0.00	3.12	Hereditary motor and sensory neuropathy, type IIc, brachyolmia type 3, metatropic dysplasia	Low expression in brain
*SERPINB9*	Serpin family B member 9	Cellular response to estrogen stimulus	0.00	−0.70	NA	Moderate expression in brain
*CNTNAP2*	Contactin associated protein‐like 2	Neuron projection development	0.00	−0.91	Cortical dysplasia‐focal epilepsy syndrome, Pitt–Hopkins like syndrome 1	High expression in brain
*CAPN11*	calpain 11	Calcium‐dependent cysteine‐type endopeptidase activity	0.00	−0.82	NA	Low expression in brain
*DNAH17*	dynein, axonemal, heavy chain 17	Cilium‐dependent cell motility	NA	NA	NA	Low expression in brain
*VPS13C*	Vacuolar protein sorting 13 homolog C	Negative regulation of parkin‐mediated stimulation of mitophagy in response to mitochondrial depolarization	0.00	−4.65	Parkinson disease	Moderate expression in brain
*UNC13B*	unc‐13 homolog B	Neurotransmitter secretion	0.00	0.51	NA	Moderate expression in brain
*SPTBN4*	Spectrin, beta, nonerythrocytic 4	Axon guidance	NA	NA	Myopathy, congenital, with neuropathy and deafness	High expression in brain
*MYOD1*	Myogenic differentiation 1	Skeletal muscle fiber development	0.00	1.96	NA	High expression in cerebellum
*MRPL15*	Mitochondrial ribosomal protein L15	Mitochondrial translational elongation	0.00	0.52	NA	Moderate expression in brain
*BTNL3*	Butyrophilin‐like protein 3	NA	0.04	1.31	NA	Low expression in brain
*TOP3B*	DNA topoisomerase 3‐beta‐1	Releases the supercoiling and torsional tension of DNA introduced during the DNA replication and transcription by transiently cleaving and rejoining one strand of the DNA duplex	0.11	3.18	NA	Moderate expression in brain
*UNK*	RING finger protein unkempt homolog	Sequence‐specific RNA‐binding protein which plays an important role in the establishment and maintenance of the early morphology of cortical neurons during embryonic development	0.99	3.85	NA	Moderate expression in brain

ExAC, Exome Aggregation Consortium; CADD, Combined Annotation Dependent Depletion (v1.3); REVEL, Rare Exome Variant Ensemble Learner; NA, not available.

a Based on Allen Brain Atlas, BioGPS and The Human Protein Atlas.

### 3.9 Other candidate genes found in two or more pedigrees

The strongest candidate variants (CADD_phred >20 and MutationTaster2 = disease causing ± MetaLR >0.75) were compared among all exomes from all pedigrees to identify common candidate genes. Three variants in *TRPV4* (OMIM 605427) were identified in three independent pedigrees. *TRPV4* has been associated with several medical disorders including autosomal dominant spinal muscular atrophy. However, all three variants are reported in ExAC and gnomAD at significant frequencies. The same SNV in *CAPN11* (OMIM 604822; NM_007058.3: c.425T>C, p.Leu142Pro) found in two independent pedigrees is reported once in gnomAD and has high CADD_phred (32), MetaLR (0.982) and REVEL (0.918) scores. *CAPN11* encodes calpain 11, an intracellular calcium‐dependent cysteine protease that shows highest expression in testis. One nonsense variant in *VPS13C* (OMIM 608879) was found in a single subject with BSP and a rare missense variant in *VPS13C* was found in another subject with BSP. Both of these *VPS13C* variants are predicted to be highly deleterious to protein function. Loss of VPS13C causes mitochondrial dysfunction and has been linked to autosomal recessive PD (Lesage et al., 2016). Moreover, *VPS13C* variants may increase risk for PD, in general (Foo et al., 2017), and, dystonia may share genetic underpinnings with PD (LeDoux et al., 2016). Other candidate genes (*SPTBN4* [OMIM 606214], *MRPL15* [OMIM 611828], *UNC13B* [605836], and *MYOD1* [159970]) shared by two pedigrees show moderate‐to‐high expression in motor regions of brain. Mice carrying recessive loss‐of‐function *Sptbn4* mutations manifest ataxia, motor neuropathy, deafness and tremor (Parkinson et al., 2001).

### 3.10 DYT13 and DYT21 loci

Within the DYT13 locus (Chr1), three subjects harbored *ATP13A2* (OMIM 610513) variants. Subject 10012 was found to have a missense variant (rs151117874, CADD_phred = 22.4, REVEL = 0.497, MetaLR = 0.8657, gnomAD = 21/272174 [3.67E‐06], Data S1). Less deleterious synonymous (CADD_phred = 17.53) and missense (CADD_phred = 21.1) variants were found in subjects 10076 and 25069, respectively (Table 1, Data S1). Recessive mutations in *ATP13A2* have been linked to Kufor–Rakeb syndrome (Ramirez et al., 2006) and spastic paraplegia 78 (Estrada‐Cuzcano et al., 2017), both of which may include dystonia as a clinical manifestation. Variants in *ATP13A2* may also contribute to oligogenic inheritance in PD (Lubbe et al., 2016). In subject 10035, a deleterious variant within the DYT21 (Chr2) locus was identified in *IMP4* (OMIM 612981; rs146322628, CADD_phred = 29.3, MetaLR = 0.83, REVEL = 0.606, gnomAD = 5.1E‐04, Data S1), and deleterious variants in *UBR4* (OMIM 609890; rs748114415, CADD_phred = 23.3, REVEL = 0.188, MetaLR = 0.46, MutationTaster2 = 0.81 [disease causing], gnomAD = 5.1E‐04, Data S1), and *ARHGEF19* (OMIM 612496; rs144638812, CADD_phred = 22.7, MetaLR = 0.64, REVEL = 0.11, MutationTaster2 = 0.55 [disease causing], gnomAD = 2.3E‐04, Data S1) were identified in the DYT13 (Chr1) locus. To date, *IMP4* and *ARHGEF19* have not been linked to a medical disorder. IMP4 interacts with the U3 snoRNA complex and is involved in nucleolar function (Granneman et al., 2003). A missense variant in *UBR4* (p.Arg5091His) was found to segregate with episodic ataxia in a large Irish pedigree (Conroy et al., 2014). UBR4 is expressed at high levels in cerebellar Purkinje cells (Allen Brain Atlas), interacts with calmodulin, colocalizes with ITPR1, and may be involved in Purkinje cell calcium homeostasis (Conroy et al., 2014). *ARHGEF19* shows significant expression in cerebellar Purkinje cells (Allen Brain Atlas) and zebrafish *arhgef19* is involved in neural tube closure (Miles et al., 2017).

## 4 DISCUSSION

The molecular and cellular mechanisms underlying BSP and other anatomical distributions of isolated dystonia remain fragmentary. Accordingly, treatments for BSP are entirely symptomatic (Pirio Richardson et al., 2017). Most commonly, BSP patients are treated with injections of botulinum toxin although, in some series, almost 50% report minimal improvement, no improvement or worsening of BSP after injections of botulinum toxins (Fernandez et al., 2014). Identification of genetic etiologies for BSP may permit development of targeted disease‐modifying therapeutics. In this study, we used exome sequencing to explore genetic contributions to BSP and provide a foundation for future case–control studies of this important focal dystonia.

Although we do provide data suggesting potential roles for *CACNA1A, REEP4, TOR2A, ATP2A3, HS1BP3/GNA14*,* DNAH17*,* TRPV4*,* CAPN11*,* VPS13C*,* UNC13B*,* SPTBN4*,* MYOD1*, and *MRPL15* in the pathogenesis of BSP, the limitations of our work should be bordered. First, we did not identify a common cosegregating genetic etiology in more than one pedigree. This points to the likely genetic heterogeneity of BSP but also suggests that one or more variants identified herein cosegregated by chance alone. Unfortunately, none of our pedigrees were powered to generate LOD (logarithm [base 10] of odds) scores >3 thereby precluding the usage of linkage analysis for validation of cosegregating variants. Second, several of the candidate variants identified with WES are reported in population databases (ExAC and gnomAD) with MAFs near the minimal population prevalence of BSP. On the other hand, noted MAFs are significantly lower than the maximal population prevalence of BSP with corrections for the markedly reduced penetrance characteristic of isolated dystonia. Furthermore, BSP and premonitory increased blinking may be much more common in the general population than commonly accepted (Conte et al., 2017). Thirdly, our genetically heterogeneous cohort included Polish, Italian, Caucasian–American and African–American pedigrees, possibly reducing the probability of detecting variants shared among pedigrees and singletons. Accordingly, follow‐up case–control analysis of individual variants identified herein will require careful attention to population stratification and large sample sizes to confidently determine if variants in candidate genes are enriched in BSP. Fourth, our prioritization of variants was predominantly driven by *in silico* predictions of deleteriousness and many potentially pathogenic candidate variants were not confirmed with Sanger sequencing or subjected to cosegregation analysis. Fifth, WES will miss most repeat expansions and does not access the mitochondrial genome. In this regard, repeat expansions are a common cause of late‐onset neurological disease and mitochondrial mutations may include dystonia as part of a more expansive neurological phenotype (LeDoux, 2012). Furthermore, our approach to CNV analysis was largely insensate to smaller structural variants such as single exonic deletions. Despite these limitations, our findings are compatible with common themes in dystonia research (calcium signaling, Purkinje cells, and dopaminergic signaling), point out potential genetic common ground with PD and ET, suggest a role for oligogenic inheritance in BSP, and provide motivation for treating a subset of BSP patients with acetazolamide.

*CACNA1A* is highly expressed in the cerebellum, particularly the Purkinje cell layer. Mutations in several genes related to calcium signaling and homeostasis and expressed in Purkinje cells have been causally associated with dystonia in humans and mice (LeDoux, 2011). In fact, virtually all genes associated with dystonia in spontaneous mutants (*tottering*,* stargazer*,* ophisthotonus*,* ducky*,* lethargic*,* waddles*, and *wriggle*) are involved in Purkinje cell Ca^2+^ signaling (*Canca1a, Cacng2, Itpr1, Cacna2d2, Cacnb4*, and *Pmca2*). In humans, autosomal‐recessive mutations in *HPCA* (OMIM 142622) cause childhood‐onset dystonia and the encoded protein, hippocalcin, is robustly expressed in Purkinje cells and serves as a Ca^2+^ sensor (Charlesworth et al., 2015; Tzingounis, Kobayashi, Takamatsu, & Nicoll, 2007). SVs in *CACNA1A* have been associated with a variety of neurological disorders including episodic ataxia type 2, familial hemiplegic migraine, spinocerebellar ataxia type 6 (SCA6), and various anatomical distributions of dystonia such as benign paroxysmal torticollis of infancy and BSP (Naik, Pohl, Malik, Siddiqui, & Josifova, 2011; Sethi & Jankovic, 2002; Shin, Douglass, Milunsky, & Rosman, 2016; Spacey, Materek, Szczygielski, & Bird, 2005; Thomsen et al., 2008). A notable percentage of patients with dystonia due to mutations in *CACNA1A* show significant improvement with acetazolamide (Spacey, 1993; Spacey et al., 2005). Unfortunately, our pedigree was lost to follow‐up and none of the affected family members were treated with acetazolamide. The α‐1 subunit of P/Q type, voltage‐dependent, calcium channel harbors a polyglutamine expansion in its C‐terminal intracellular domain and the novel missense variant p.Pro2421Val identified in our pedigree with BSP is near this expansion (Figure 3). In contrast, the previously described BSP‐variant was likely associated with nonsense‐mediated decay and haploinsufficiency (Spacey et al., 2005). Mutations linked to familial hemiplegic migraine appear to operate via gain‐of‐function mechanisms whereas the SCA6 polyglutamine repeat and loss‐of‐function mutations may lead to neuronal cell death (Cain & Snutch, 2011). In this context, it is worthy to note that reduced Purkinje cell density was found in two individuals with BSP and cervical dystonia (Prudente et al., 2013).

REEP4 is a microtubule‐binding endoplasmic reticulum and nuclear envelope protein (Schlaitz, Thompson, Wong, Yates, & Heald, 2013). Depletion of REEP4 from HeLa cells is associated with defective cell division and proliferation of intranuclear membranes derived from the nuclear envelope (Schlaitz et al., 2013). Similarly, omega‐shaped nuclear blebs have been used as a phenotypic measure of torsinA (encoded by *TOR1A*) dysfunction (Laudermilch et al., 2016). In *Xenopus*, loss of REEP4 causes defects of nervous system development and paralysis of embryos (Argasinska et al., 2009). Mutations in *REEP1* (OMIM 609139) and *REEP2* (OMIM 609347) are associated with spastic paraplegia (SPG) types 31 (SPG31), and 72 (SPG72). Although dystonia is not a clinical feature typically reported in SPG31 and SPG72 cases, dystonia is not uncommon in several other SPGs, including SPG7, SPG15, SPG26, SPG35, and SPG47 (van Gassen et al., 2012; Klebe, Stevanin, & Depienne, 2015).

A ΔGAG deletion in Exon 5 of *TOR1A* was the first SV to be linked to isolated dystonia (Ozelius et al., 1997). TorsinA interacts with LAP1, a transmembrane protein ubiquitously expressed in the inner nuclear membrane. Recessive mutations of *TOR1AIP1* (OMIM 614512) which encodes LAP1 are associated with severe early‐onset generalized dystonia and progressive cerebellar atrophy (Dorboz et al., 2014). Another torsinA interacting protein, torsin family 2 member A (encoded by *TOR2A*) was found to harbor a missense variant in one of our pedigrees with BSP. Similar to the ΔGAG mutation in *TOR1A*, the penetrance of the p.Arg190Cys missense variant identified in our pedigree was less than 50%. *TOR2A* is a member of the human torsin gene family (Laudermilch et al., 2016; Ozelius et al., 1999). *TOR1A*,* TOR2A* and *TOR1AIP1* all show relatively high expression in cerebellar Purkinje cells (Allen Brain Atlas).

A nonsynonymous SNV in *ATP2A3* (NM_005173.3: c.1966C>T, p.Arg656Cys) was found in five definitely‐affected subjects from a discordant pedigree with BSP from Italy. However, this variant was not detected in one possibly affected family member with writer's cramp. This could be either a phenocopy or evidence against the causality of *ATP2A3*. Furthermore, the p.Arg656Cys variant is present at notably high frequency in gnomAD (183/276,114 alleles, no homozygotes, 0.13% of 138,057 subjects). BSP is the most common focal dystonia in Italy with a crude prevalence rate of 133 per million or 0.013%. Even with a penetrance of <20%, this suggests that p.Arg656Cys may not be pathogenic or, at least, pathogenic in isolation, requiring digenic inheritance of another pathogenic variant. On the other hand, p.Arg656Cys is predicted to be highly deleterious, may contribute to other anatomical distributions of dystonia, and, like *ATP1A3*, could be involved in the etiopathogenesis of other neurological disorders such as Parkinson disease, Alzheimer disease, and brain tumors (Kawalia et al., 2017; Korosec, Glavac, Volavsek, & Ravnik‐Glavac, 2009; Matak et al., 2016). In this regard, *ATP2A3* shows striking expression in cerebellar Purkinje cells and dopaminergic neurons of the substantia nigra pars compacta (Allen Brain Atlas). *ATP2A3* encodes a sarcoplasmic/endoplasmic reticulum Ca^2+^ ATPase and disorders of Purkinje cell (LeDoux, 2011) and dopaminergic (Surmeier, Halliday, & Simuni, 2017) calcium homeostasis have been linked to dystonia and Parkinson disease, respectively.

A small pedigree (Figure 6) with BSP+ and Parkinsonism harboring variants in *HS1BP3* and *GNA14* highlights the distinct possibility of oligogenic inheritance in BSP and other anatomical distributions of dystonia. In particular, all of the exomes sequenced in this study harbored more than one potentially pathogenic variant. Since most of our pedigrees were small and moderate numbers of variants showed *in silico* evidence of deleteriousness, we did not assess cosegregration for all of the identified candidate variants. However, we determined that both *GNA14* and *HS1PB3* were attractive candidate genes. Guanine nucleotide‐binding protein subunit alpha‐14 (encoded by *GNA14*) interacts with dynein, axonemal, light chain 4 (UniProt) which is expressed at high levels in sperm and brain. *GNA14* appears to play a key role in the genetic architecture underlying normal gray matter density (Chen et al., 2015) and a *GNA14* deletion mutation has been reported in a patient with early‐onset Alzheimer disease (Lazarczyk et al., 2017). *HS1BP3* shows moderate expression in brain (The Human Protein Atlas), and, in cerebellum, appears at highest levels in Purkinje cells (Allen Brain Atlas). Multipoint linkage analysis in four large pedigrees with ET identified a critical region between loci D2S2150 and D2S220 on Chr 2p which includes *HS1BP3* (Higgins, Loveless, Jankovic, & Patel, 1998). The p.A265G HCLS1‐binding protein 3 (HS1BP3) variant encoded by *HS1BP3* is in linkage disequilibrium with ET but is unlikely to be causal since it is present at high frequency in the general population (Shatunov et al., 2005). It remains unknown if other coding or noncoding variants in *HS1BP3* are causally related to the pathogenesis of ET. HS1BP3 negatively regulates auto‐phagy (Holland et al., 2016), a cellular pathway closely tied to several neurodegenerative disorders including PD (Nash, Schmukler, Trudler, Pinkas‐Kramarski, & Frenkel, 2017). In this regard, ET and PD may be related to adult‐onset dystonia through common genetics (De Rosa et al., 2016; Dubinsky, Gray, & Koller, 1993; Hedera et al., 2010; LeDoux et al., 2016; Louis et al., 2012; Straniero et al., 2017).

Oligenic inheritance is caused by mutations in two or more proteins with a functional relationship through direct interactions, membership in a pathway, or coexpression in a specific cell type. Given that functional groups of genes tend to colocalize within chromosomes (Thevenin, Ein‐Dor, Ozery‐Flato, & Shamir, 2014), the possibility of oligogenic inheritance of variants found within a locus defined by linkage analysis cannot be ignored. Our focused analyses of the DYT13 and DYT21 loci provide genes and variants for cosegregation analysis in these previously detailed dystonia pedigrees and suggest that digenic or higher‐order oligogenic inheritance of variants within a disease‐associated locus may be causal in some pedigrees and isolated cases with BSP. In this context, cosegregating variants in *CIZ1* and *SETX* were linked to cervical dystonia in a large American pedigree (Xiao et al., 2012).

Blepharospasm exerts important effects on health‐related quality of life (Hall et al., 2006). Many patients with BSP experience annoying dry eye symptoms and photophobia (Hallett, Evinger, Jankovic, Stacy, & Workshop, 2008). Oral medications such as anticholinergics and benzodiazepines are mildly beneficial in some subjects. Many patients with BSP show moderate benefit from injections of botulinum toxin. However, injections are expensive, painful and may be denied by third‐party payers. Although deep brain stimulation has been used to treat some individuals with BSP+ phenotypes, responses have been mixed (Reese et al., 2011). Major advances in the treatment of BSP demand a deeper understanding of its genetic etiopathogenesis. Our work provides a platform for follow‐up case–control analyses of identified variants, evaluation of digenic and higher‐order oliogenic etiologies for BSP (Deltas, 2017), and generation of animal models to help assess the pathogenicity of identified variants. Future work will demand attention to the effects of genetic background, oligogenic inheritance, pleiotropy, confounds of phenocopies, and the limitations of WES.

## CONFLICT OF INTEREST

None declared.

## WEB RESOURCES

1000 Genomes, http://www.1000genomes.org/
Allen Brain Atlas, http://www.brain-map.org/
BioGRID, https://thebiogrid.org/
BioGPS, http://biogps.org/
ExAC Browser, http://exac.broadinstitute.org/
CADD, http://cadd.gs.washington.edu/
Clustal Omega, https://www.ebi.ac.uk/Tools/msa/clustal0/
gnomAD, http://gnomad.broadinstitute.org/
MARRVEL, http://marrvel.org/
MutationTaster, http://www.mutationtaster.org/
NCBI, https://www.ncbi.nlm.nih.gov/
NHLBI Exome Sequencing Project (ESP) Exome Variant Server (EVS), http://evs.gs.washington.edu/EVS/
OMIM, http://www.omim.org/
UniProt, http://www.uniprot.org/
