# Supplementary material for: Whole‐exome sequencing for variant discovery in blepharospasm
Source: Mol Genet Genomic Med. 2018 May 16;6(4):601–26. doi: 10.1002/mgg3.411 (PMC6081235; doi:10.1002/mgg3.411)
Supplement: Supplementary file 2 [file MGG3-6-601-s002.pdf]

Supplemental Data

Table S1. Primers for Sanger sequencing, qPCR and digital PCR

Primer	Sequence (5’ →3’)	Locus	Usage	Product (bp)
**TOR2A_E3F**	ggtttgggaagaggtctggt	NC_000009.12 127733630 - 611	Sanger	505
**TOR2A_E3R**	ggaaaacttcttgacctggca	NC_000009.12 127733126 - 146		
**ATP2A3_E14F**	ctgagcaggtggtaggtcag	NC_000017.11 3941368 - 349	Sanger	479
**ATP2A3_E14R**	ggagagttgcacggcttg	NC_000017.11 3940890 - 907		
**MYH13_E30F**	tggcaaaattggtgaagacag	NC_000017.11 10313477 - 457	Sanger	375
**MYH13_E30R**	tcccttggcttgtgtcctc	NC_000017.11 10313103- 121		
**REEP4_E3F**	ctcctgtccagccatgtgt	NC_000008.11 22140382-364	Sanger	533
**REEP4_E4R**	agcagcaggactgggataga	NC_000008.11 22139850-869		
**GTDC1_E4F**	taatgggataagcctattttgaa	NC_000002.11 144208945 - 923	Sanger	415
**GTDC1_E4R**	gatggagaagcaacatcctca	NC_000002.11 144208531 - 551		
**PCDH15_E12F**	ttcaaatggccctttaacgta	NC_000010.11 54196032 - 012	Sanger	430
**PCDH15_E12R**	ccatatctttgtcatatttcccatt	NC_000010.11 54195603 - 627		
**PITPNM1_E22F**	cgtggtcaggtaggagttgc	NC_000011.9 67261167 - 148	Sanger	264
**PITPNM1_E22R**	acattgccttctggcgtagt	NC_000011.9 67260904 - 923		
**SLC6A11_E7F**	tttcagagaccagggctacc	NC_000003.11 10953700 - 719	Sanger	225
**SLC6A11_E7R**	tctcagggtttggtggagag	NC_000003.11 10953924 - 905		
**TRPV4_E8F**	aatgcagctgaggaatggat	NC_000012.11 110232426 - 407	Sanger	380
**TRPV4_E8R**	ggacccagaaggatgaggtt	NC_000012.11 110232047 - 066		
**ZFYVE9_E11F**	gggaatgtggtgagcaactt	NC_000001.10 52759124 - 143	Sanger	377
**ZFYVE9_E11R**	ttatgcccaatacacgcaga	NC_000001.10 52759500 - 481		
**PCDHA2_E1F**	gctgcttgattctcggtttc	NC_000005.9 140174987 - 5006	Sanger	300
**PCDHA2_E1R**	actgattgggcaaaagttgg	NC_000005.9 140175286 - 5267		
**INO80_E11F**	gagcttgctttggctactgg	NC_000015.9 41365911 - 892	Sanger	345
**INO80_E11R**	aaggcttgtggggtatttcc	NC_000015.9 41365567 - 586		
**IGSF21_E9F**	ttccttccctgagctctgaa	NC_000001.10 18703825 - 844	Sanger	227
**IGSF21_E9R**	aatgacctgcctgaggtgtc	NC_000001.10 18704051 - 032		
**MYOD1_E1F**	acgtgaggacgagcatgtg	NC_000011.9 17741554 - 572	Sanger	445
**MYOD1_E1R**	ccgccctaacttcctcactt	NC_000011.9 17741998 - 979		
**SERPINB9-E5F**	tcatccaatgcatcaggttc	NC_000006.11 2893849 - 830	Sanger	223
**SERPINB9-E5R**	ttaaaaagcttcccccacct	NC_000006.11 2893627 - 646		
**CNTNAP2_E11F**	atattgcccagacagcttgg	NC_000007.13 147182942 - 961	Sanger	401
**CNTNAP2_E11R**	agtcctttgccaccagagaa	NC_000007.13 147183342 - 323		
**CAPN11_E5F**	tggggtacagggtcaagttc	NC_000006.11 44139935 - 954	Sanger	378
**CAPN11_E5R**	gatttctttggtgggcagat	NC_000006.11 44140312 - 293		
**WDFY3_E19F**	ctggataatgatcttggcactt	NC_000004.11 85718041 - 020	Sanger	391
**WDFY3_E19R**	tggcttccttgcaaatttct	NC_000004.11 85717651 - 670		
**NEFH_E1F**	ctcaggccatgatgagcttc	NC_000022.10 29876244 - 263	Sanger	449
**NEFH_E1R**	ctcgtacagctcgcccatag	NC_000022.10 29876692 - 673		
**MYT1L_E9F**	aggaggaggagatcgaggag	NC_000002.11 1946867 - 848	Sanger	499
**MYT1L_E9R**	aacactgttttgccctttgc	NC_000002.11 1946369 - 388		
**HS1BP3_E1F**	gcagtgaccactgaggacag	NC_000002.11 20845402 - 383	Sanger	406
**HS1BP3_E1R**	aagcacttgctctggatgct	NC_000002.11 20844997 - 5016		
**AGAP1_E2F**	ttgaggctgatgagaacgtg	NC_000002.11 236625990 - 6009	Sanger	434
**AGAP1_E2R**	aagggtttttaccgcttggt	NC_000002.11 236626423 - 6404		
**EPS15L1_E15F**	cccatggtcacaggtcctac	NC_000019.9 16524847 - 828	Sanger	333
**EPS15L1_E15R**	agggagggaagttttcctga	NC_000019.9 16524515 - 534		
**SCN1A_E1F**	gctcatgtttcatgacaagaattt	NC_000002.11 166930262 -239	Sanger	452
**SCN1A_E1R**	gtccaaggaatgcagtaggc	NC_000002.11 166929811 - 830		
**LRP1B_E20F**	tatcatttgagccccaggag	NC_000002.11 141708160 - 141	Sanger	537
**LRP1B_E20R**	ccaaagggaaaaagcaaaga	NC_000002.11 141707624 - 643		
**PCDHGA3_E1F**	ctccaacactggcgtcctat	NC_000005.9 140725124 - 143	Sanger	564
**PCDHGA3_E1R**	gccaccaccaggtacagagt	NC_000005.9 140725687 - 668		
**LAMA1_E34F**	tgcaaaggatggcagtgtag	NC_000018.9 6995642 - 623	Sanger	461
**LAMA1_E34R**	agaaactggcagggtcaaaa	NC_000018.9 6995182 - 201		
**CADPS_E2F**	tctcacatttccctctttttctg	NC_000003.11 62751692 - 670	Sanger	458
**CADPS_E2R**	agcctcagcctcttcatcag	NC_000003.11 62751235 - 254		
**SNPH_E6F**	cgtggacatcaacatccaga	NC_000020.11 1305070 - 089	Sanger	486
**SNPH_E6R**	aggatggtgtcaaggtcagg	NC_000020.11 1305555 - 536		
**ATP2BA_E20F**	tgcaagttccactgcattaca	NC_000012.11 89985206 - 186	Sanger	411
**ATP2BA_E20R**	ctggggatgaagaggtagca	NC_000012.11 89984796 - 815		
**SLC12A2_E17F**	ccctgtgccttagctattcc	NC_000005.9 127497147 - 166	Sanger	499
**SLC12A2_E17R**	agccaggtgagccaaagtag	NC_000005.9 127497645 - 626		

**LRP1_E21F**	gagttggtgcttccaaggag	NC_000012.11 57566788 - 807	Sanger	428
**LRP1_E21R**	ctttgccgctactctgctct	NC_000012.11 57567215 - 196		
**LRP1_E21F**	gagttggtgcttccaaggag	NC_000012.11 57566788 - 807	Sanger	428
**LRP1_E21R**	ctttgccgctactctgctct	NC_000012.11 57567215 - 196		
**GCH1_E5F**	agctggtgtgtcttggctct	NC_000014.8 55312699 - 680	Sanger	352
**GCH1_E5R**	aggctcagggatggaaatct	NC_000014.8 55312348 - 367		
**DDHD2_E2F**	taaagcttccacccatccag	NC_000008.10 38090329 - 348	Sanger	452
**DDHD2_E2R**	tgaagaggggagagtctttgtc	NC_000008.10 38090780 - 759		
**HECW2_E14F**	ttctggtgcttctgcatgtc	NC_000002.11 197157533 - 514	Sanger	403
**HECW2_E14R**	tcacaaacaacctgctgtcc	NC_000002.11 197157131 - 150		
**CDH4_E16F**	ctactcggaaatgcccttca	NC_000020.10 60511627 - 646	Sanger	443
**CDH4_E16R**	ctctgctcagtcctgctcct	NC_000020.10 60512069 - 050		
**RABL2B_E2F**	actgagggaagcagtctgga	NC_000022.11 50782440 - 421	Sanger	436
**RABL2B_E2R**	cctccacctgacacacacaa	NC_000022.11 50782005 - 024		
**AP4B1_E9F**	acaccttttctgctggcact	NC_000001.10 114439198 - 179	Sanger	436
**AP4B1_E9R**	ccagacgtgactcaccaaac	NC_000001.10 114438794 - 813		
**SCN3A_E3F**	ccaatcgcttgctttcctaa	NC_000002.11 166033002 - 2983	Sanger	444
**SCN3A_E3R**	tgctgtatataaggcccagaaaa	NC_000002.11 166032559 - 2581		
**CAPN11_E5F**	tggggtacagggtcaagttc	NC_000006.11 44139935 - 954	Sanger	378
**CAPN11_E5R**	gatttctttggtgggcagat	NC_000006.11 44140312 - 293		
**REEP2_E4F**	ctaagagtgaggggctgtgc	NC_000005.9 137779997 - 80016	Sanger	312
**REEP2_E4R**	gagggccagtctgtatgagg	NC_000005.9 137780308 - 80289		
**MYO1B-E16F**	tcctgtggcttttgttattgttt	NC_000002.11 192250450 - 472	Sanger	426
**MYO1B-E16R**	gaggtacagcctccttgcag	NC_000002.11 192250875 - 856		
**ZZEF1_E5F**	gggacttctggatcctgtca	NC_000017.10 4016172 - 153	Sanger	305
**ZZEF1_E5R**	gcttttccctatcccctcag	NC_000017.10 4015868 - 887		
**KCNA5_E1F**	gtggccatcttcccctactt	NC_000012.11 5154400 - 419	Sanger	542
**KCNA5_E1R**	ctctgagtgccctgctcttc	NC_000012.11 5154941 - 922		
**KCNH4_E9F**	acctggtaggcacacaggag	NC_000017.10 40321810 -791	Sanger	446
**KCNH4_E9R**	tgggattacaggtgtgagca	NC_000017.10 40321365 - 384		
**CHRNA7_E6F**	tccatttgctctggactgtg	NC_000015.9 32449684 - 703	Sanger	446
**CHRNA7_E6R**	ttttaaagcttgcccaggaa	NC_000015.9 32450036 - 017		
**KCNG4_E3F**	ggcccaagacaagtgtcagt	NC_000016.9 84256534 - 515	Sanger	325
**KCNG4_E3R**	cacgtagaccaaaggggaga	NC_000016.9 84256210 - 229		
**PLP1_E2F**	tgctgaaagccaaaacctct	NC_000023.10 103040359 - 378	Sanger	358
**PLP1_E2R**	tgggagggcaggtacttaca	NC_000023.10 103040716 - 697		
**KCNS1_E4F**	cctgtgcgacgactacgac	NC_000020.10 43727143 - 125	Sanger	411
**KCNS1_E4R**	atggagacgcagctgaagag	NC_000020.10 43726733 - 752		
**ACLY_E20F**	ccttgtgtccaggctgaaat	NC_000017.10 40039615 -596	Sanger	317
**ACLY_E20R**	gattacctggttcgcctcaa	NC_000017.10 40039299 - 318		
**TRPV4_E5F**	tctacgtcctgcaccctacc	NC_000012.11 110238652 - 633	Sanger	312
**TRPV4_E5R**	tgcagacaccaaccagtatca	NC_000012.11 110238343 - 363		
**TBP_E3F**	accccattattctccgaagg	NC_000006.11 170870760 - 779	Sanger	555
**TBP_E3R**	gcggtacaatcccagaactc	NC_000006.11 170871314 - 295		
**HK1_E12F**	ctcagtccagctgttgtgga	NC_000010.10 71142201 - 220	Sanger	454
**HK1_E12R**	gtgcatcaccagtgtgaagg	NC_000010.10 71142654 - 635		
**PRUNE2_E9F**	gttctctgaactcggcttgg	NC_000009.11 79318543 - 524	Sanger	506
**PRUNE2_E9R**	aaattgaaggggctttttgg	NC_000009.11 79318038 - 057		
**NUMBL_E10F**	cctgtccctctcctctctcc	NC_000019.9 41174065 - 046	Sanger	409
**NUMBL_E10R**	ccatctgtgagggtgtgatg	NC_000019.9 41173657 - 676		
**MRPL15_E2F**	tgtgtggagctttgagcaac	NC_000008.10 55048914 - 933	Sanger	358
**MRPL15_E2R**	taatggaaattggggacctg	NC_000008.10 55049271 - 252		
**MRPL15_E4F**	gcactttggagggaaaaaca	NC_000008.10 55055123 - 142	Sanger	315
**MRPL15_E4R**	accaacatttgccattcact	NC_000008.10 55055437 - 418		
**MYOD1_E1F**	ccgcctgagcaaagtaaatg	NC_000011.9 17741689 - 708	Sanger	310
**MYOD1_E1R**	ccgccctaacttcctcactt	NC_000011.9 17741998 - 979		
**SPTBN4_E12F**	ccaggacaactttgggtatga	NC_000019.9 41009733 - 753	Sanger	571
**SPTBN4_E12R**	ccgtgtccagcatgtttcta	NC_000019.9 41010303 - 284		
**UNC13B_E39F**	ttgcagatatgcgtgaagga	NC_000009.11 35403782 - 801	Sanger	351
**UNC13B_E39R**	gcctcttgactgcaaagacc	NC_000009.11 35404132 - 113		
**UNC13B_E36F**	gggctgaagaaaaccttcct	NC_000009.11 35400317 - 336	Sanger	221
**UNC13B_E36R**	ctcccaagggcatgaagata	NC_000009.11 35400527 - 508		
**VPS13C_E69F**	gaaatgcgcctccctattc	NC_000015.9 62174911 - 893	Sanger	327
**VPS13C_E69R**	tgcaggctaatcgatcaaaa	NC_000015.9 62174585 - 604		
**VPS13C_E83F**	tgacagaaggggaatttttga	NC_000015.9 62148838 - 818	Sanger	413
**VPS13C_E83R**	tgcaaaacagtctaaaagcaatg	NC_000015.9 62148426 - 448		
**DNAH17_E60F**	taaaggggagagggaaagaaa	NC_000017.10 76456242 - 223	Sanger	357
**DNAH17_E60R**	ctcagggatgtgctccttgt	NC_000017.10 76455886 - 905		
**DNAH17_E81F**	cttactggcagcacacctga	NC_000017.10 76420278 - 259	Sanger	472
**DNAH17_E81R**	cacctcttccacaccaacct	NC_000017.10 76419807 - 826		

**CAPN11_E5F**	gagaatggggtacagggtca	NC_000006.11 44139930 - 949	Sanger	344
**CAPN11_E5R**	caaaacccaccctaataaatgc	NC_000006.11 44140273 - 252		
**LRRC49_18F**	ggagcactggaatctctgga	NC_000015.9 71300818 - 837	Digital PCR	76
**LRRC49_18R**	tgaaagtgaaggagactgttgtg	NC_000015.9 71300893 - 871		
**GOLGA8A_82F**	tcagactctcagagctaacac	NC_000015.9 34678416 - 438	Digital PCR	72
**GOLGA8A_82R**	aagccacctctcctttttgg	NC_000015.9 34678487 - 468		
**BTNL3_76F**	cctgtcctcaggctggttc	NC_000005.9 180424277 - 295	Digital PCR	76
**BTNL3_76R**	tcctgtccttgtggaccttt	NC_000005.9 180424336 - 317		
**CLEC18B_76F**	ggtcaacgggaagacaatca	NC_000016.9 74447044 - 025	Digital PCR	84
**CLEC18B_76R**	gctttgaagcagcctgagac	NC_000016.9 74446961 - 980		
**CYP2A7_16F**	ttgaggaccggaccaagat	NC_000019.9 41383228 - 210	Digital PCR	80
**CYP2A7_16R**	actcatggggatcacgtctc	NC_000019.9 41383149 - 168		
**LILRA3_7F**	agccatcactctcagtgcag	NC_000019.9 54802767 - 748	Digital PCR	72
**LILRA3_7R**	cagagccacactggaaggtc	NC_000019.9 54802696 - 715		
**RRP7A_67F**	gactgaggcagccagctt	NC_000022.10 42910218 - 201	Digital PCR	86
**RRP7A_67R**	gctgccaggcgtagaagtt	NC_000022.10 42910133 - 151		
**SLC2A14_85F**	tctctcctccctgtcctctgt	NC_000012.11 7985439 - 419	Digital PCR	74
**SLC2A14_85R**	gccgattgtagcaactgtga	NC_000012.11 7985366 - 385		
**SLC2A3_83F**	ttgtcaacctgttggctgtc	NC_000012.11 8084064 - 045	Digital PCR	77
**SLC2A3_83R**	gatcagcatttcaaccgactt	NC_000012.11 8083988 - 008		
**TOP3B_17F**	ctaggcgagcctgaccac	NC_000022.10 22324664 - 647	Digital PCR	79
**TOP3B_17R**	tgaatgcacagccgattc	NC_000022.10 22324586 - 603		
**UNK_19F**	ccctgacctcaagcatctct	NC_000017.10 73814754 - 773	Digital PCR	105
**UNK_19R**	gcagagcgtttgcattcat	NC_000017.10 73814858 - 840		
**PDPK1_1F**	gtggccctgtgtcctgag	NC_000016.9 2647016 - 033	qPCR	117
**PDPK1_1R**	aaataaaccctgacgccaaa	NC_000016.9 2647132 - 113		
**MAPT_16F**	agtgtgggcaccttcatcc	NC_000017.10 44049493 - 511	qPCR	102
**MAPT_16R**	gtgggaggacaatcgtatgg	NC_000017.10 44049594 - 575		
**GABRG1_54F**	aaattcatttattattgtgctcagatg	NC_000004.11 46043295 - 269	qPCR	72
**GABRG1_54R**	attattcattggaatcagagtggat	NC_000004.11 46043224 - 248		
**GABRA2_51E**	ccaagctagacggacacaaag	NC_000004.11 46253283 - 263	qPCR	69
**GABRA2_51R**	tccattatcacaaactcttgtctga	NC_000004.11 46253215 - 239		
**GABRA4_25F**	gggctactgggaagttgtca	NC_000004.11 46930426 - 407	qPCR	66
**GABRA4_25R**	ttgtgccagatccagaaggt	NC_000004.11 46930361 - 380		
**VPS41_5F**	gagaaggaggtggagtctatgc	NC_000007.13 38858295 - 274	qPCR	61
**VPS41_5R**	cctccatctttggagctcat	NC_000007.13 38858235 - 254		
**NSF_33F**	cagtgggattatggcaacct	NC_000017.10 44805852 - 871	qPCR	67
**NSF_33R**	aagcaaccctcctgcctaa	NC_000017.10 44805913 - 895		
**PDXDC1_9F**	cccccttttcctttcaggtc	NC_000016.9 15122841 - 859	qPCR	78
**PDXDC1_9R**	cagttcccatacacgagcac	NC_000016.9 15122918 - 899		
**TPPP_14F**	agggtttatgccccttgc	NC_000005.9 665534 - 517	qPCR	91
**TPPP_14R**	agttgcgagccaggtgag	NC_000005.9 665444 - 461		
**TCAF1_67F**	gaaagtctcctttgcaccctta	NC_000007.13 143549606 - 585	qPCR	78
**TCAF1_67R**	ctttcctggttttcatcctca	NC_000007.13 143549529 - 549		
**UNK_86F**	cggctaatgatggtgagtacc	NC_000017.10 73808430 - 450	qPCR	60
**UNK_86R**	gggcaagaaccagagtgc	NC_000017.10 73808489 - 472		

Table S2. WES variants examined with Sanger sequencing and co-segregation analyses

Pedigree	Subject	Gene	Phenotype	BSP Family History	Sequencing result	Co-segregation
**10012**	10012	*KCNH4*	BSP	No	c.1505T>A (NM_012285)	NA
**10012**	10012	*CHRNA7*	BSP	No	c.584_585delTG (NM_001190455.2)	NA
**10012**	10012	*SPTBN4*	BSP	No	c.1543C>T (NM_020971.2)	NA
**10014**	10014	*KCNG4*	BSP	No	c.1045C>T (NM_172347.2)	NA
**10014**	10014	*PLP1*	BSP	No	c.64A>G (NM_000533)	NA
**10014**	10014	*KCNS1*	BSP	No	c.434A>G (NM_002251.4)	NA
**10014**	10014	*ACLY*	BSP	No	c.2227A>AG (NM_001096)	NA
**10014**	10014	*VPS13C*	BSP	No	c.10954C>T (NM_020821.2)	NA
**10035**	10035	*TRPV4*	BSP	No	c.769C>G (NM_021625.4)	NA
**10035**	10035	*TBP*	BSP	No	c.223_224delCA (NM_003194)	NA
**10036**	10036	*HK1*	BSP	No	c.1422A>G (NM_001322364.1)	NA
**10036**	10036	*PRUNE2*	BSP	No	c.8151delG (NM_015225)	NA
**10036**	10036	*NUMBL*	BSP	No	c.1323delC (NM_004756.4)	NA
**10036**	10036	*MRPL15*	BSP	No	c.485_498delTAGC TATTGCTGCC (NM_014175.3)	NA
**10076**	10076	*CAPN11*	BSP	No	c.425T>C (NM_007058.3)	NA
**10076**	10076	*REEP2*	BSP	No	c.217G>A (NM_016606)	NA
**10076**	10076	*MYO1B*	BSP	No	c.1462C>T (NM_012223.4)	NA
**10076**	10076	*DNAH17*	BSP	No	c.425T>C (NM_007058.3)	NA
**10076**	10076	*CAPN11*	BSP	No	c.13295G>A (NM_173628.3)	NA
**10043**	10043-I-1	*GNA14*	BSP	Yes	c.989_990delCA (NM_004297.3)	Yes
**10043**	10043-I-2	*GNA14*	Normal	Yes	No variant	Yes
**10043**	10043-II-1	*GNA14*	Normal	Yes	No variant	Yes
**10043**	10043-II-2	*GNA14*	BSP	Yes	c.989_990delCA (NM_004297.3)	Yes
**10043**	10043-I-1	*CCZ1*	BSP	Yes	No variant	No
**10043**	10043-I-2	*CCZ1*	Normal	Yes	No variant	No
**10043**	10043-II-1	*CCZ1*	Normal	Yes	No variant	No
**10043**	10043-II-2	*CCZ1*	BSP	Yes	No variant	No
**10043**	10043-I-1	*RWDD2A*	BSP	Yes	c.76C>T (NM_033411.4)	Yes
**10043**	10043-I-2	*RWDD2A*	Normal	Yes	No variant	Yes
**10043**	10043-II-1	*RWDD2A*	Normal	Yes	c.76C>T (NM_033411.4)	Yes
**10043**	10043-II-2	*RWDD2A*	BSP	Yes	c.76C>T (NM_033411.4)	Yes
**10043**	10043-I-1	*NEFH*	BSP	Yes	c.338A>T (NM_033411)	Yes
**10043**	10043-I-2	*NEFH*	Normal	Yes	No variant	Yes

**10043**	10043-II-1	*NEFH*	Normal	Yes	No variant	Yes
**10043**	10043-II-2	*NEFH*	BSP	Yes	c.338A>T (NM_033411)	Yes
**10043**	10043-I-1	*HS1BP3*	BSP	Yes	c.94C>A (NM_022460.3)	Yes
**10043**	10043-I-2	*HS1BP3*	Normal	Yes	No variant	Yes
**10043**	10043-II-1	*HS1BP3*	Normal	Yes	No variant	Yes
**10043**	10043-II-2	*HS1BP3*	BSP	Yes	c.94C>A (NM_022460.3)	Yes
**10064**	10064	*HECW2*	BSP	Yes	c.2896G>T (NM_020760.3)	NA
**10064**	10064	*CDH4*	BSP	Yes	No variant	NA
**10064**	10064	*RABL2B*	BSP	Yes	c.91G>A (NM_007081.2)	NA
**10064**	10064	*AP4B1*	BSP	Yes	c.1345A>T (NM_001253852.2)	NA
**10064**	10064	*SCN3A*	BSP	Yes	c.127delT (NM_006922)	NA
**10178**	10178	*ZZEF1*	BSP	Yes	c.1025G>A (NM_015113.3)	NA
**10178**	10178	*KCNA5*	BSP	Yes	c.1327A>G (NM_002234.3)	NA
**10178**	10178	*MRPL15*	BSP	Yes	c.201delT (NM_014175.3)	NA
**10178**	10178	*MYOD1*	BSP	Yes	c.485C>T (NM_002478.4)	NA
**10193**	10193-II-5	*IGSF21*	BSP	Yes	c.1300C>T (NM_032880.4)	Yes
**10193**	10193-III-3	*IGSF21*	Normal	Yes	c.1300C>T (NM_032880.4)	Yes
**10193**	10193-II-5	*MYOD1*	BSP	Yes	c.485C>T (NM_002478.4)	Yes
**10193**	10193-III-3	*MYOD1*	Normal	Yes	c.485C>T (NM_002478.4)	Yes
**10274**	10274-II-3	*SLC6A11*	BSP	Yes	c.903T>A (NM_014229.2)	No
**10274**	10274-II-6	*SLC6A11*	BSP	Yes	c.903T>A (NM_014229.2)	No
**10274**	10274-II-2	*SLC6A11*	BSP	Yes	No variant	No
**10274**	10274-II-4	*SLC6A11*	Normal	Yes	No variant	No
**10274**	10274-II-1	*SLC6A11*	Normal	Yes	c.903T>A (NM_014229.2)	No
**10274**	10274-II-3	*TRPV4*	BSP	Yes	c.1337G>T (NM_021625.4)	Yes
**10274**	10274-II-6	*TRPV4*	BSP	Yes	c.1337G>T (NM_021625.4)	Yes
**10274**	10274-II-2	*TRPV4*	BSP	Yes	c.1337G>T (NM_021625.4)	Yes
**10274**	10274-II-4	*TRPV4*	Normal	Yes	No variant	Yes
**10274**	10274-II-1	*TRPV4*	Normal	Yes	No variant	Yes
**10274**	10274-II-3	*WDFY3*	BSP	Yes	c.3145G>C (NM_014991.4)	Yes
**10274**	10274-II-6	*WDFY3*	BSP	Yes	c.3145G>C (NM_014991.4)	Yes
**10274**	10274-II-2	*WDFY3*	BSP	Yes	c.3145G>C (NM_014991.4)	Yes
**10274**	10274-II-4	*WDFY3*	Normal	Yes	No variant	Yes
**10274**	10274-II-1	*WDFY3*	Normal	Yes	No variant	Yes
**10274**	10274-II-3	*ZFYVE9*	BSP	Yes	c.3224T>C (NM_004799.3)	Yes
**10274**	10274-II-6	*ZFYVE9*	BSP	Yes	c.3224T>C (NM_004799.3)	Yes

**10274**	10274-II-2	*ZFYVE9*	BSP	Yes	c.3224T>C (NM_004799.3)	Yes
**10274**	10274-II-4	*ZFYVE9*	Normal	Yes	No variant	Yes
**10274**	10274-II-1	*ZFYVE9*	Normal	Yes	c.3224T>C (NM_004799.3)	Yes
**10455**	10455	*CADPS*	BSP	Yes	c.544T>CT (NM_003716)	NA
**10455**	10455	*SNPH*	BSP	Yes	c.619C>T (NM_014723.3)	NA
**10455**	10455	*ATP2B1*	BSP	Yes	c.3542C>A (NM_001682.2)	NA
**10455**	10455	*SLC12A2*	BSP	Yes	c.2512G>A (NM_001046.2)	NA
**10455**	10455	*CAPN11*	BSP	Yes	c.425T>C (NM_007058.3)	NA
**10455**	10455	*SPTBN4*	BSP	Yes	c.1543C>T (NM_020971.2)	NA
**10455**	10455	*VPS13C*	BSP	Yes	c.9605C>G (NM_020821.2)	NA
**10908**	10908-II-3	*CHST2*	BSP	Yes	c.728A>G (NM_004267.4)	No
**10908**	10908-II-1	*CHST2*	BSP	Yes	c.728A>G (NM_004267.4)	No
**10908**	10908-II-4	*CHST2*	Normal	Yes	No variant	No
**10908**	10908-II-6	*CHST2*	Normal	Yes	c.728A>G (NM_004267.4)	No
**10908**	10908-II-5	*CHST2*	BSP	Yes	c.728A>G (NM_004267.4)	No
**10908**	10908-III-4	*CHST2*	BSP	Yes	No variant	No
**10908**	10908-II-7	*CHST2*	BSP	Yes	No variant	No
**10908**	10908-III-12	*CHST2*	BSP	Yes	No variant	No
**10908**	10908-III-9	*CHST2*	BSP	Yes	c.728A>G (NM_004267.4)	No
**10908**	10908-II-2	*CHST2*	Normal	Yes	c.728A>G (NM_004267.4)	No
**10908**	10908-II-8	*CHST2*	Normal	Yes	c.728A>G (NM_004267.4)	No
**10908**	10908-III-1	*CHST2*	Normal	Yes	No variant	No
**10908**	10908-II-3	*IFT172*	BSP	Yes	c.728A>G (NM_004267.4)	No
**10908**	10908-II-1	*IFT172*	BSP	Yes	c.728A>G (NM_004267.4)	No
**10908**	10908-II-4	*IFT172*	Normal	Yes	No variant	No
**10908**	10908-II-6	*IFT172*	Normal	Yes	c.728A>G (NM_004267.4)	No
**10908**	10908-II-5	*IFT172*	BSP	Yes	c.728A>G (NM_004267.4)	No
**10908**	10908-III-4	*IFT172*	BSP	Yes	No variant	No
**10908**	10908-II-7	*IFT172*	BSP	Yes	No variant	No
**10908**	10908-III-12	*IFT172*	BSP	Yes	No variant	No
**10908**	10908-III-9	*IFT172*	BSP	Yes	c.728A>G (NM_004267.4)	No
**10908**	10908-II-2	*IFT172*	Normal	Yes	c.728A>G (NM_004267.4)	No
**10908**	10908-II-8	*IFT172*	Normal	Yes	c.728A>G (NM_004267.4)	No
**10908**	10908-III-1	*IFT172*	Normal	Yes	No variant	No
**10908**	10908-II-3	*PCDHA2*	BSP	Yes	c.547C>CT (NM_018905)	No
**10908**	10908-II-1	*PCDHA2*	BSP	Yes	c.547C>CT (NM_018905)	No

**10908**	10908-II-4	*PCDHA2*	Normal	Yes	c.547C>CT (NM_018905)	No
**10908**	10908-II-6	*PCDHA2*	Normal	Yes	No variant	No
**10908**	10908-II-5	*PCDHA2*	BSP	Yes	c.547C>CT (NM_018905)	No
**10908**	10908-III-4	*PCDHA2*	BSP	Yes	No variant	No
**10908**	10908-II-7	*PCDHA2*	BSP	Yes	c.547C>CT (NM_018905)	No
**10908**	10908-III-12	*PCDHA2*	BSP	Yes	c.547C>CT (NM_018905)	No
**10908**	10908-III-9	*PCDHA2*	BSP	Yes	c.547C>CT (NM_018905)	No
**10908**	10908-II-2	*PCDHA2*	Normal	Yes	c.547C>CT (NM_018905)	No
**10908**	10908-II-8	*PCDHA2*	Normal	Yes	c.547C>CT (NM_018905)	No
**10908**	10908-III-1	*PCDHA2*	Normal	Yes	c.547C>CT (NM_018905)	No
**10908**	10908-II-3	*PITPNM1*	BSP	Yes	c.3242C>T (NM_004910.2)	No
**10908**	10908-II-1	*PITPNM1*	BSP	Yes	c.3242C>T (NM_004910.2)	No
**10908**	10908-II-4	*PITPNM1*	Normal	Yes	No variant	No
**10908**	10908-II-6	*PITPNM1*	Normal	Yes	c.3242C>T (NM_004910.2)	No
**10908**	10908-II-5	*PITPNM1*	BSP	Yes	c.3242C>T (NM_004910.2)	No
**10908**	10908-III-4	*PITPNM1*	BSP	Yes	c.3242C>T (NM_004910.2)	No
**10908**	10908-II-7	*PITPNM1*	BSP	Yes	c.3242C>T (NM_004910.2)	No
**10908**	10908-III-12	*PITPNM1*	BSP	Yes	No variant	No
**10908**	10908-III-9	*PITPNM1*	BSP	Yes	c.3242C>T (NM_004910.2)	No
**10908**	10908-II-2	*PITPNM1*	Normal	Yes	No variant	No
**10908**	10908-II-8	*PITPNM1*	Normal	Yes	No variant	No
**10908**	10908-III-1	*PITPNM1*	Normal	Yes	c.3242C>T (NM_004910.2)	No
**10908**	10908-II-3	*REEP4*	BSP	Yes	c.109C>T (NM_025232.3)	Yes
**10908**	10908-II-1	*REEP4*	BSP	Yes	c.109C>T (NM_025232.3)	Yes
**10908**	10908-II-4	*REEP4*	Normal	Yes	c.109C>T (NM_025232.3)	Yes
**10908**	10908-II-6	*REEP4*	Normal	Yes	No variant	Yes
**10908**	10908-II-5	*REEP4*	BSP	Yes	c.109C>T (NM_025232.3)	Yes
**10908**	10908-III-4	*REEP4*	BSP	Yes	c.109C>T (NM_025232.3)	Yes
**10908**	10908-II-7	*REEP4*	BSP	Yes	c.109C>T (NM_025232.3)	Yes
**10908**	10908-III-12	*REEP4*	BSP	Yes	c.109C>T (NM_025232.3)	Yes
**10908**	10908-III-9	*REEP4*	BSP	Yes	c.109C>T (NM_025232.3)	Yes
**10908**	10908-II-2	*REEP4*	Normal	Yes	No variant	Yes
**10908**	10908-II-8	*REEP4*	Normal	Yes	No variant	Yes
**10908**	10908-III-1	*REEP4*	Normal	Yes	No variant	Yes
**45263**	45263-II-1	*CNTNAP2*	BSP	Yes	c.1723A>T (NM_014141.5)	No

**45263**	45263-II-3	*CNTNAP2*	BSP	Yes	No variant	No
**45263**	45263-II-1	*INO80*	BSP	Yes	c.1384G>A (NM_017553)	Yes
**45263**	45263-II-3	*INO80*	BSP	Yes	c.1384G>A (NM_017553)	Yes
**45263**	45263-II-1	*DNAH17*	BSP	Yes	c.9473C>T (NM_173628.3)	Yes
**45263**	45263-II-3	*DNAH17*	BSP	Yes	c.9473C>T (NM_173628.3)	Yes
**85020**	85020	*LRP1*	BSP	Yes	c.3208C>T (NM_002332.2)	NA
**85020**	85020	*GCH1*	BSP	Yes	c.610C>T (NM_000161)	NA
**85020**	85020	*DDHD2*	BSP	Yes	c.87_88insA (NM_015214)	NA
**NG0362**	NG0362-II-2	*CACNA1A*	BSP	Yes	c.7261_7262delinsG (NM_001127222.1)	Yes
**NG0362**	NG0362-I-1	*CACNA1A*	BSP	Yes	c.7261_7262delinsG (NM_001127222.1)	Yes
**NG0362**	NG0362-III-1	*CACNA1A*	BSP	Yes	c.7261_7262delinsG (NM_001127222.1)	Yes
**NG0362**	NG0362-II-3	*CACNA1A*	Normal	Yes	No variant	Yes
**NG0362**	NG0362-I-2	*CACNA1A*	Normal	Yes	No variant	Yes
**NG0362**	NG0362-III-2	*CACNA1A*	Normal	Yes	c.7261_7262delinsG (NM_001127222.1)	Yes
**NG0369**	NG0369-II-2	*TOR2A*	BSP	Yes	c.568C>T (NM_130459.3)	Yes
**NG0369**	NG0369-III-2	*TOR2A*	BSP	Yes	c.568C>T (NM_130459.3)	Yes
**NG0369**	NG0369-III-6	*TOR2A*	BSP	Yes	c.568C>T (NM_130459.3)	Yes
**NG0369**	NG0369-III-4	*TOR2A*	Normal	Yes	No variant	Yes
**NG0369**	NG0369-I-2	*TOR2A*	Normal	Yes	c.568C>T (NM_130459.3)	Yes
**NG0369**	NG0369-II-3	*TOR2A*	Normal	Yes	c.568C>T (NM_130459.3)	Yes
**NG0369**	NG0369-II-6	*TOR2A*	Normal	Yes	No variant	Yes
**NG0369**	NG0369-II-7	*TOR2A*	Normal	Yes	c.568C>T (NM_130459.3)	Yes
**NG0369**	NG0369-II-8	*TOR2A*	Normal	Yes	No variant	Yes
**NG0369**	NG0369-IV-1	*TOR2A*	Normal	Yes	No variant	Yes
**NG0369**	NG0369-IV-4	*TOR2A*	Normal	Yes	No variant	Yes
**NG0369**	NG0369-II-2	*GTDC1*	BSP	Yes	c.70C>A (NM_024659.4)	Yes
**NG0369**	NG0369-III-2	*GTDC1*	BSP	Yes	c.70C>A (NM_024659.4)	Yes
**NG0369**	NG0369-III-6	*GTDC1*	BSP	Yes	c.70C>A (NM_024659.4)	Yes
**NG0369**	NG0369-III-4	*GTDC1*	Normal	Yes	No variant	Yes
**NG0369**	NG0369-I-2	*GTDC1*	Normal	Yes	No variant	Yes
**NG0369**	NG0369-II-3	*GTDC1*	Normal	Yes	c.70C>A (NM_024659.4)	Yes
**NG0369**	NG0369-II-6	*GTDC1*	Normal	Yes	No variant	Yes
**NG0369**	NG0369-II-7	*GTDC1*	Normal	Yes	No variant	Yes
**NG0369**	NG0369-II-8	*GTDC1*	Normal	Yes	c.70C>A (NM_024659.4)	Yes
**NG0369**	NG0369-IV-1	*GTDC1*	Normal	Yes	No variant	Yes
**NG0369**	NG0369-IV-4	*GTDC1*	Normal	Yes	No variant	Yes
**NG0369**	NG0369-II-2	*PCDHA2*	BSP	Yes	No variant	No

**NG0369**	NG0369-III-2	*PCDHA2*	BSP	Yes	No variant	No
**NG0369**	NG0369-III-6	*PCDHA2*	BSP	Yes	No variant	No
**NG0369**	NG0369-III-4	*PCDHA2*	Normal	Yes	No variant	No
**NG0369**	NG0369-I-2	*PCDHA2*	Normal	Yes	No variant	No
**NG0369**	NG0369-II-3	*PCDHA2*	Normal	Yes	No variant	No
**NG0369**	NG0369-II-6	*PCDHA2*	Normal	Yes	No variant	No
**NG0369**	NG0369-II-7	*PCDHA2*	Normal	Yes	No variant	No
**NG0369**	NG0369-II-8	*PCDHA2*	Normal	Yes	No variant	No
**NG0369**	NG0369-IV-1	*PCDHA2*	Normal	Yes	No variant	No
**NG0369**	NG0369-IV-4	*PCDHA2*	Normal	Yes	No variant	No
**NG0450**	NG0450-IV-3	*CNTNAP2*	BSP	Yes	c.653C>T (NM_014141.5)	Yes
**NG0450**	NG0450-V-4	*CNTNAP2*	BSP	Yes	c.653C>T (NM_014141.5)	Yes
**NG0450**	NG0450-V-6	*CNTNAP2*	BSP	Yes	c.653C>T (NM_014141.5)	Yes
**NG0450**	NG0450-IV-2	*CNTNAP2*	Normal	Yes	c.653C>T (NM_014141.5)	Yes
**NG0450**	NG0450-V-7	*CNTNAP2*	Normal	Yes	c.653C>T (NM_014141.5)	Yes
**NG0450**	NG0450-IV-3	*TRPV4*	BSP	Yes	c.745T>A (NM_001177431.1)	Yes
**NG0450**	NG0450-V-4	*TRPV4*	BSP	Yes	c.745T>A (NM_001177431.1)	Yes
**NG0450**	NG0450-V-6	*TRPV4*	BSP	Yes	c.745T>A (NM_001177431.1)	Yes
**NG0450**	NG0450-IV-2	*TRPV4*	Normal	Yes	c.745T>A (NM_001177431.1)	Yes
**NG0450**	NG0450-V-7	*TRPV4*	Normal	Yes	c.745T>A (NM_001177431.1)	Yes
**NG0450**	NG0450-IV-3	*SERPINB9*	BSP	Yes	c.565C>T (NM_004155.5)	Yes
**NG0450**	NG0450-V-4	*SERPINB9*	BSP	Yes	c.565C>T (NM_004155.5)	Yes
**NG0450**	NG0450-V-6	*SERPINB9*	BSP	Yes	c.565C>T (NM_004155.5)	Yes
**NG0450**	NG0450-IV-2	*SERPINB9*	Normal	Yes	c.565C>T (NM_004155.5)	Yes
**NG0450**	NG0450-V-7	*SERPINB9*	Normal	Yes	c.565C>T (NM_004155.5)	Yes
**NG1072**	NG1072-II-5	*ATP2A3*	BSP	Yes	c.1966C>T (NM_005173.3)	Yes
**NG1072**	NG1072-IV-2	*ATP2A3*	BSP	Yes	c.1966C>T (NM_005173.3)	Yes
**NG1072**	NG1072-II-2	*ATP2A3*	BSP	Yes	c.1966C>T (NM_005173.3)	Yes
**NG1072**	NG1072-III-5	*ATP2A3*	BSP	Yes	c.1966C>T (NM_005173.3)	Yes
**NG1072**	NG1072-IV-3	*ATP2A3*	BSP	Yes	c.1966C>T (NM_005173.3)	Yes
**NG1072**	NG1072-III-1	*ATP2A3*	Possible	Yes	No variant	Yes
**NG1072**	NG1072-III-2	*ATP2A3*	Possible	Yes	c.1966C>T (NM_005173.3)	Yes
**NG1072**	NG1072-III-4	*ATP2A3*	Normal	Yes	No variant	Yes
**NG1072**	NG1072-III-7	*ATP2A3*	Normal	Yes	c.1966C>T (NM_005173.3)	Yes
**NG1072**	NG1072-IV-1	*ATP2A3*	Normal	Yes	No variant	Yes
**NG1072**	NG1072-IV-4	*ATP2A3*	Normal	Yes	c.1966C>T (NM_005173.3)	Yes

**NG1072**	NG1072-II-3	*ATP2A3*	Normal	Yes	c.1966C>T (NM_005173.3)	Yes
**NG1072**	NG1072-II-4	*ATP2A3*	Normal	Yes	No variant	Yes
**NG1072**	NG1072-II-6	*ATP2A3*	Normal	Yes	c.1966C>T (NM_005173.3)	Yes
**NG1072**	NG1072-II-5	*MYH13*	BSP	Yes	c.4054G>A (NM_003802.2)	No
**NG1072**	NG1072-IV-2	*MYH13*	BSP	Yes	c.4054G>A (NM_003802.2)	No
**NG1072**	NG1072-II-2	*MYH13*	BSP	Yes	c.4054G>A (NM_003802.2)	No
**NG1072**	NG1072-III-5	*MYH13*	BSP	Yes	c.4054G>A (NM_003802.2)	No
**NG1072**	NG1072-IV-3	*MYH13*	BSP	Yes	No variant	No
**NG1072**	NG1072-III-1	*MYH13*	Possible	Yes	No variant	No
**NG1072**	NG1072-III-2	*MYH13*	Possible	Yes	c.4054G>A (NM_003802.2)	No
**NG1072**	NG1072-III-4	*MYH13*	Normal	Yes	No variant	No
**NG1072**	NG1072-III-7	*MYH13*	Normal	Yes	No variant	No
**NG1072**	NG1072-IV-1	*MYH13*	Normal	Yes	No variant	No
**NG1072**	NG1072-IV-4	*MYH13*	Normal	Yes	No variant	No
**NG1072**	NG1072-II-3	*MYH13*	Normal	Yes	c.4054G>A (NM_003802.2)	No
**NG1072**	NG1072-II-4	*MYH13*	Normal	Yes	No variant	No
**NG1072**	NG1072-II-6	*MYH13*	Normal	Yes	No variant	No

Table S3. Evaluation of potential CNVs with qPCR of gDNA

Patient ID	Gene	hg19 CNV Coordinates	Log2 Ratio	Probes	Normal controls (N = 8) mean ± SEM	Fold change
**10908-II-3**	*NSF*	Chr17: 44701604-44806343	-0.38	17	1.00 ± 0.07	0.67
**25056**	*PDXDC1*	Chr16: 15068591-15128400	-0.33	30	1.00 ± 0.07	0.71
**85020**	*UNK*	Chr17: 73808156-73820465	0.58	17	1.00 ± 0.02	1.35
**NG0450-V-4**	*PDPK1*	Chr16: 2611444-2647772	-0.42	15	1.04 ± 0.12	2.77
**NG0450-V-4**	*MAPT*	Chr17: 44049195-44101476	-0.50	20	1.00 ± 0.02	1.35
**NG0450-V-6**	*GABRG1*	Chr4: 46043019-46125922	-0.53	11	1.00 ± 0.03	0.93
**NG0450-V-6**	*GABRA2*	Chr4: 46252386-46390771	-0.53	13	1.00 ± 0.03	0.89
**NG0450-V-6**	*GABRA4*	Chr4: 46930270-46979196	-0.53	6	1.00 ± 0.02	0.92
**NG0450-V-6**	*VPS41*	Chr7: 38857346-38948925	-1.0	10	1.01 ± 0.04	1.14
**45263**	*TPPP*	Chr5: 665231-678082	-0.43	4	1.01 ± 0.06	1.21
**45263**	*TCAF1*	Chr7: 143549362-143557592	-0.45	6	1.02 ± 0.07	0.96
